# Toward Reliable Spectroscopic Analysis of Reaction Kinetics in Polaritonic Chemistry

**DOI:** 10.1002/nap2.70143

**Published:** 2026-05-27

**Authors:** Robrecht M. A. Vergauwe, J. Jussi Toppari, Gerrit Groenhof

**Affiliations:** ^1^ Nanoscience Center and Department of Chemistry University of Jyväskylä Jyväskylä Finland; ^2^ Nanoscience Center and Department of Physics University of Jyväskylä Jyväskylä Finland

**Keywords:** polaritonic chemistry, reaction kinetics, spectroscopy, transfer matrix method

## Abstract

Recent reports suggest that chemical reaction rates can change inside an optical cavity. These effects have been attributed to the hybridization of molecular vibrational modes with cavity modes into polaritons, but the underlying mechanism remains debated. Recently, attempts to reproduce the key experiments have sometimes failed, which also poses ambiguity and impedes the determination of the possible mechanism. Without a reliable theoretical framework, polaritonic chemistry—seeking to use optical resonators as catalysts for reactions—has reached a pivotal stage. Standardized protocols for reproducible cavity experiments are therefore urgently needed. Here, we identify pitfalls in approaches that monitor reaction progress with UV/Vis spectroscopy. Using the transfer‐matrix method, we analyze a model pseudo‐first‐order reaction and assess how transient cavity thickness variations, cavity inhomogeneity, and fitting protocols influence the extracted rate constant. We find that changes in cavity thickness upon reactant introduction can strongly distort apparent kinetics when monitoring at a single wavelength, an artifact that is mitigatable by spectral smoothing. Additionally, we demonstrate that, unlike in many studies, the asymptotic extinction should be treated as a fitting parameter rather than fixed to the final experimental value. By identifying these pitfalls, our work lays the foundation for more robust analyses and reliable measurements in polaritonic chemistry.

## Graphical abstract

Vibrational strong coupling has been reported to modify chemical reaction rates, yet its mechanism remains unresolved. We expose pitfalls in UV/Vis‐based kinetic analyses and emphasize the need for standardized cavity protocols.

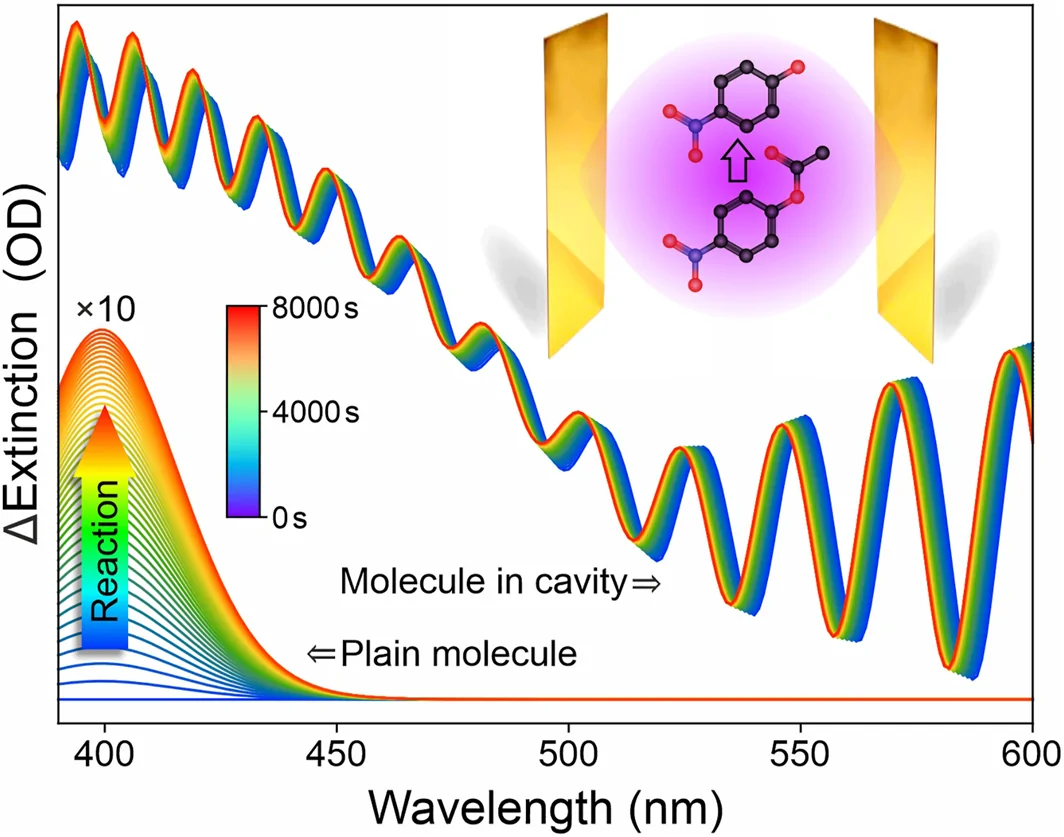

## 1 Introduction

It is possible to create hybrid states of light and matter, known as polaritons, using an IR‐active vibration of an organic substance and a photonic cavity—a structure that confines electromagnetic fields of a certain frequency in a limited region in space [1, 2, 3]. If (1) the cavity frequency matches the energy of the vibration, that is, resonance occurs, and (2) the molecule‐cavity EM fields interaction, that is, photon absorption/re‐emission, is stronger than any other loss mechanism such as photon leakage or dissipation by the molecule, the so‐called strong coupling regime is reached, and the wavefunctions of the molecule and cavity field hybridize, forming the polaritons [4, 5]. This phenomenon is called vibrational strong coupling (VSC). Because this entails a direct modification of the molecular wavefunction, the question arises if such hybridization could affect the physico‐chemical properties of the system.

To address this question, several groups have begun measuring these properties [1, 2, 3]. These endeavors have produced fascinating claims, such as reaction rate acceleration [6, 7, 8, 9] and decelerations [10, 11, 12, 13, 14, 15], chemical equilibrium shifts [16, 17, 18, 19, 20, 21], and the transformation of an insulator into a conductor [22], raising both excitement and controversy [23, 24, 25]. Yet the central mystery remains unresolved: how, and to what extent, does VSC actually control chemical reactions? Existing theoretical models explain fragments of the observations [26, 27, 28, 29, 30] but fail to provide predictive power [31, 32, 33].

On the experimental side, the role of photonic cavities and polaritons in catalysis remains highly debated. George and coworkers initially reported a tenfold acceleration of the fluoride‐mediated cleavage of *para*‐nitrophenol acetate (PNPA) under vibrational strong coupling (VSC) [7] and a 10,000 ‐fold acceleration of cyanide hydrolysis [6]. However, these results could not be reproduced by others [8, 24, 25], nor by the same authors, who later observed only a 2.5 – 3 ‐fold rate increase—much smaller and within the typical uncertainties of kinetic measurements [8]. Gómez Rivas and colleagues likewise found only a very modest rate change in a similar cavity setup, though they reported a 2.7‐fold enhancement when the reaction was coupled to plasmonic nanoparticle arrays [9]. These discrepancies place polaritonic chemistry at a pivotal stage, underscoring the need for careful experiments to resolve the current controversies.

Most of the polaritonic chemistry experiments have been performed in microfluidic Fabry–Pérot (FP) micro‐cavities, built from commercial IR transmission cells with μm optical path lengths [1, 10, 14, 34]. An ideal Fabry–Pérot structure consists of two planar mirrors facing each other in a perfectly parallel manner and supports a series of regularly spaced transmission modes. In practice, microfluidic VSC cavities have thin ∼10 nm Au mirrors and display clear transmission modes in the infrared. In the UV/Vis, these cavities have only an oscillatory transmission spectrum of higher order cavity modes enveloped by the imprint of the intraband transitions of Au [8, 12, 24]. Recently, Michon and Simpkins demonstrated that optical measurements of organic dyes in such nonideal Fabry–Pérot cavities can give rise to artifacts because of the higher order mode fringes in the UV/Vis transmission spectra. [35]. A dye with a peak at 600 nm will display a shifted peak position when there is sufficient broadening due to cavity width inhomogeneity, that is, the cavity has a range of different cavity widths in the area being probed, for example, due to misalignment or bending of the mirrors under pressure [35]. Such width inhomogeneity can have important implications for reaction kinetic measurements on (pro‐)chromophores such as PNPA, and one cannot assume anymore that chemical kinetics in a Fabry–Pérot structure can be directly extracted from UV/Vis data, in particular if only a single wavelength is analyzed.

In addition to cavity width inhomogeneity, characterized by a static distribution of cavity widths, we identified another issue during our VSC chemistry experiments: Microfluidic VSC cavities display almost always some degree of contraction after injection, that is, the cavity width does not remain fixed but instead decreases during the course of the reaction measurement. The impact of this contraction on reaction rate estimation has not yet been investigated. Given that a residual cavity mode structure is visible in cavity UV/Vis spectra, failing to account for spectral effects of transient cavity contraction can also impact the reaction rate estimation. The unavoidable presence of cavity width distributions and temporal cavity width changes—static and dynamic phenomena, respectively—forces us to consider the extent to which reaction rates can be reliably estimated from Fabry–Pérot UV/Vis data.

To address these issues, we introduce a controllable model system for systematically investigating how various optical artifacts influence a (pseudo‐)first‐order reaction of a PNPA‐type pro‐chromophore using transfer matrix method (TMM) simulations. Beyond static cavity‐width inhomogeneity, the model includes dynamic cavity contraction—motivated by experimental observations—as well as different procedures for processing raw absorption spectra and fitting reaction rates, including the determination of the asymptotic extinction, ${\text{Ext}}_{\infty }$, which is shown to be of critical importance. We focus on (pseudo‐)first‐order kinetics, as this regime predominates in studies of bimolecular reactions (A+B→C) in physical and polaritonic chemistry. Since our model excludes any coupling between molecular vibrations and the cavity field, any apparent changes in the reaction rate inferred from the simulated spectroscopic signals must arise solely from optical artifacts or analysis procedures. Our simulations reveal that cavity thickness fluctuations upon reactant introduction can significantly distort kinetic traces when monitoring the reaction progress at a single wavelength. Although spectral smoothing and integration can mitigate this effect, care must be taken in interpreting the resulting kinetics. We also show that the asymptotic extinction should be treated as a fitting parameter rather than fixed to the final experimental value. Finally, we discuss how the artifacts identified here may have influenced prior analyses of PNPA hydrolysis under VSC conditions.

## 2 Materials and Methods

Fabry–Pérot structures are simulated using the standard transfer matrix method (TMM) on the following stack structure: air (n=1)/Au mirror/medium layer/Au mirror/air (n=1) [36]. The two Au mirrors layers are modeled as perfectly planar 10 nm thick Au layers with a complex refractive index taken from Rosenblatt et al. [37]. The medium layer thickness is 5 μm, except when simulating the experiments of Lather et al., Singh et al., and Weisehan et al., in which the width was 18–22 μm [7, 8, 24] (see SI). To model a reaction that produces a *para*‐nitrophenolate‐type chromophore within the organic medium layer, the time‐dependent dielectric function of this layer was constructed from a fully Kramers–Kronig compatible Gaussian broadened Lorentz oscillator model, $\epsilon_{\text{absorber}}\left(\omega \right)$ [38], parameterized for an absorption peak at 400 nm with a linewidth of 1000 ${\text{cm}}^{-1}$ at full‐width at half maximum (FWHM, see Supplementary Information (SI) for more details), and a background refractive index $n_{r}$ of 1.3, similar to methanol and ethanol. The amplitude factor of this model, $\tilde{a}=1.2\times 10^{-4}$, was multiplied with a coefficient $0\leq c_{r}\left(t\right)\leq 1$ that reflects the progression of the (pseudo‐)first order reaction with rate $k_{\text{reaction}}$:

(1) $$\epsilon_{\text{medium}}\left(\omega ,t\right)=\epsilon_{0}+c_{r}\left(t\right)\;\cdot \;\tilde{a}\;\cdot \;\epsilon_{\text{absorber}}\left(\omega \right)$$

with

(2) $$c_{r}\left(t\right)=1-e^{-k_{\text{reaction}}t}.$$

Thus, the time‐dependent concentration of the reaction product C(t) is proportional to $c_{r}\left(t\right)\;\cdot \;\tilde{a}$.

Unless specified otherwise, we use the reaction rate of $k_{\text{reaction}}=2\times 10^{-4}{\text{s}}^{-1}$ and the simulations were carried out for 8000 s with time steps of 10 s. The time‐dependent transmission spectra $T_{\text{sim}}\left(\lambda ,t\right)$, defined as the fraction of power transmitted, were computed for the wavelengths λ from 390 to 850 nm with a sampling resolution of 1 nm (see SI for comment on light polarization). The time‐dependent cavity extinction spectra for unpolarized light in units of optical density (OD) were subsequently computed as follows:

(3) $$\text{Ext}\left(\lambda ,t\right)=-\log_{10}\left(T_{\text{sim}}\left(\lambda ,t\right)\right)$$

This way, we mimic previous VSC experiments, in which the reaction progress was tracked using commercial UV/Vis spectrophotometers with unpolarized light sources.

To calculate the reference time‐dependent UV/Vis extinction spectrum of a reaction taking place in a standard transmission measurement cell, a TMM calculation is performed on a stack lacking the Au mirrors: air (n=1)/medium layer/air (n=1). The reaction and the calculation of the cell extinction spectra were treated identically to the procedure described above for the cavity.

To account for cavity width inhomogeneity in our simulations, we followed the approach of Michon and Simpkins [35] and assumed that the probe beam samples a Gaussian distribution of cavity widths, with a standard deviation given by $\sigma_{\text{FWHM}}=L_{\text{FWHM}}/\left(2\sqrt{2\;\ln\;2}\right)$, where $L_{\text{FWHM}}$ denotes the full width at half maximum of the distribution. The widths, $L_{i}$, were then deterministically sampled from this distribution, and the transmission spectrum of the Fabry–Péot structure was computed as a weighted sum of the spectra corresponding to the individual cavity widths (see SI):

$$T=\frac{1}{N}\sum_{i}^{N}w\left(L_{i}\right)\times T\left(L_{i}\right)$$

with $T\left(L_{i}\right)$ being the transmission of a cavity with width $L_{i}$, and weight

$$w\left(L_{i}\right)=\frac{1}{\sqrt{2\pi }\sigma_{\text{FWHM}}}\;\cdot \;\exp\left[\frac{-{\left(L-L_{i}\right)}^{2}}{2\sigma_{\text{FWHM}}^{2}}\right]$$

We computed N=17 transmission spectra, with cavity lengths evenly distributed between $L-4L_{\text{FWHM}}$ and $L+4L_{\text{FWHM}}$ around the average cavity length L. In Supporting Information (SI), we demonstrate the much higher performance, that is, reduced noise and computation cost, of the deterministic sampling of the cavity width distribution compared to random sampling (Supporting Information S1; Figure S12).

We tested four different techniques for extracting the reaction progress from the calculated extinction spectra (for details see SI).

1. Savitzky–Golay filtering [39] on a 51‐data point domain.
2. Rolling‐average filtering on a domain of 41 points.
3. Integration of the spectra over spectral regions between 405–415, 395–425, or 400–450 nm. The integrals were divided by the length of the spectral region to facilitate direct comparison.
4. Integration after rolling‐average filtering. Integration over the spectral region between 410 and 430 nm after the application of a rolling‐average filter (2).

The progress of the reaction was either inferred from the differential extinction at 410 nm with respect to the extinction at time 0, that is, ΔExt(410nm,t)=Ext(410nm,t)−Ext(410nm,0s) (Equation 3) or from the integrated differential extinction spectrum. All the filtering/integration/averaging were done to the original extinction spectra, that is, before the subtraction of Ext(410nm,t=0s).

Direct fitting of the reaction progress to the differential‐extinction time traces was performed using one of the two exponential models:

(4) $$\Delta \text{Ext}\left(t\right)={\text{Ext}}_{\infty }\left(1-e^{-k_{\text{fit}}t}\right),$$

or

(5) $$\begin{array}{ll} \Delta \text{Ext}(t) & ={\text{Ext}}_{0}\left[1-e^{-k_{\text{fit}}\left(t-t_{0}\right)}\right]+{\text{Ext}}_{\text{bck}}\\ \;{\text{Ext}}_{\infty } & ={\text{Ext}}_{0}+{\text{Ext}}_{\text{bck}}. \end{array}$$

Here, $k_{\text{fit}}$ is the fitted rate constant; any deviation from the expected reaction rate $k_{\text{reaction}}$ therefore indicates an artifact rather than a physical change in kinetics. Equation (4) is used for simulations of an ideal, homogeneous cavity (Figure 1 and Supporting Information S1; Figure S1), where neither time delays nor background extinctions are present. When cavity contraction is included (Figure 2, Supporting Information S1; Figures S5 and S6), the more general four‐parameter form (Equation 5) is employed. This expression allows the exponential to be translated in both time and extinction via the parameters $t_{0}$ and ${\text{Ext}}_{\text{bck}}$ and corresponds to the model commonly used to analyze experimental data, where finite response times and background signals are unavoidable. Importantly, our simulations do not contain such effects—the reaction begins at t=0 with zero initial product concentration. We nevertheless use the full model to evaluate how spectral changes induced by cavity contraction during the measurement could bias the fit and thereby distort the extracted rate constant inside the cavity.

In addition to direct exponential fitting, the simulated reaction traces were also analyzed using a standard linearization approach used in chemical kinetics. Specifically, we computed $\ln\left[{\text{Ext}}_{\infty }-\text{Ext}\left(t\right)\right]$ and fitted the resulting trace with a linear function. For most datasets, the value of ${\text{Ext}}_{\infty }$ used in this transformation was obtained from the preceding exponential fits. When Equation (4) is used, ${\text{Ext}}_{\infty }$ is one of the two fit parameters. When Equation (5) is applied, ${\text{Ext}}_{\infty }$ equals ${\text{Ext}}_{0}+{\text{Ext}}_{\text{bck}}$.

Complete details of the simulations reproducing previously published experiments are provided in Supporting Information. The SI also contains a full description of our own VSC chemistry experiments, including the procedures and analyses related to the observed cavity contraction.

## 3 Results and Discussion

### 3.1 Rate in an Ideal Cavity

We begin by examining the simplest scenario: an ideal Fabry–Pérot cavity formed by two perfectly parallel mirrors separated by an absorbing layer. The layer contains a Gaussian‐broadened absorption band centered at 400 nm, representing a *para*‐nitrophenolate‐type (PNP) chromophore (Figure 1 and Supporting Information S1; Figure S1). This model is chosen to emulate previous VSC catalysis experiments on PNPA, as well as our own ongoing studies of PNPA hydrolysis under VSC conditions.

**FIGURE 1:**
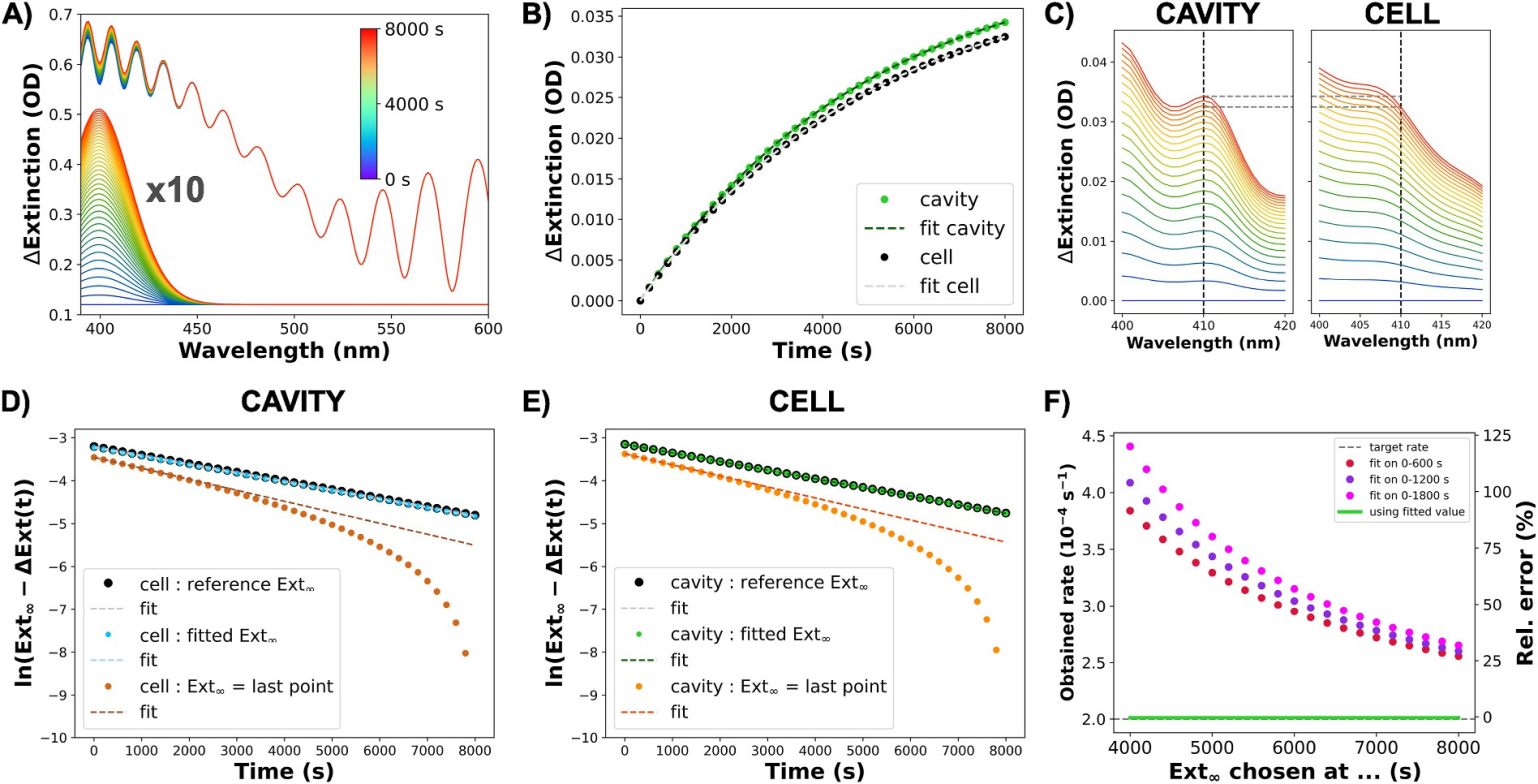
First order kinetics analysis in an ideal VSC cavity. (A) Simulated UV/Vis extinction spectra of ideal Fabry–Pérot cavities with perfectly parallel 10 nm thick Au mirrors held at a constant distance of 5 μm together with the extinction spectra of the absorbing layer between the mirrors (multiplied 10‐fold for clarity). The Gaussian extinction of the 5 μm thick absorption layer increases exponentially with constant rate $k_{\text{reaction}}$ over time (dark blue → dark red). Extinction parameters: peak = 400 nm, $\sigma_{\text{gauss}}=1000$ ${\text{cm}}^{-1}$, $\tilde{a}=1.2\times 10^{-4}$, $k_{\text{reaction}}=2\times 10^{-4}$ $s^{-1}$. (B) Analysis of reaction time trace, that is, extinction at 410 nm as a function of time for both cavity (green) and cell (no mirrors—black) with exponential fits. (C) Comparison of the differential extinction spectra over time of an ideal cavity (left) and cell (right). The double dashed lines indicates where ΔExt(8000s) is in the cavity and cell, respectively. (D) Analysis of linearized reaction time traces for the cell measurement with linear fits. The three traces are calculated with an ${\text{Ext}}_{\infty }$ derived by direct computation (black), from an exponential fitting (blue) or selection of the maximum observed extinction (brown). (E) Analysis of linearized reaction time traces of a cavity setup with linear fits. The three traces are calculated with an ${\text{Ext}}_{\infty }$ derived by direct computation (black), from exponential fitting (green) or selection of the maximum observed extinction (orange). (F) Dependence of the fitted reaction rate, obtained from the linearized trace analysis using the maximum observed extinction as ${\text{Ext}}_{\infty }$, on the time a hypothetical experiment was stopped as well as on the size of the fit window used.

To simulate a (pseudo‐)first‐order chemical reaction with a fixed rate constant of $k_{\text{reaction}}=2.0\times 10^{-4}\;{\text{s}}^{-1}$, we scale the absorption intensity of the chromophore over time by modulating the dielectric function of the absorbing layer (see Methods for details). This approach produces a time‐dependent increase of the cavity extinction with the expected first‐order kinetics, as shown in Figure 1A,B.

Figure 1A shows the UV/Vis extinction spectra of the ideal cavity at various time points, together with the extinction of the absorbing layer (scaled for clarity) obtained from its time‐dependent dielectric function (full and zoomed versions are provided in Supporting Information S1; Figure S1A,B). A gradual increase in extinction in the 400–450 nm region is visible, although its amplitude remains smaller than the modulation introduced by the cavity fringes. This behavior closely resembles typical VSC chemistry experiments on PNPA reported by us and others [8, 24].

Because the cavity width is fixed in this ideal Fabry–Pérot structure, the reaction progress can be extracted straightforwardly by taking the extinction at 410 nm at t and subtracting from it the extinction at *t* = 0 s. The resulting time‐dependent change in extinction is plotted in Figure 1B for both the cavity and, for comparison, a reference structure without mirrors. Following conventions in the field, we refer to the latter as the *cell*, representing a simple short path‐length transmission geometry.

To extract the reaction rates, the differential extinction traces (Figure 1B) were analyzed by direct exponential fitting (Equation 4). This procedure yields rate constants of 2.0017 for the cavity and $2.0122\times 10^{-4}\;s^{-1}$ for the cell, with goodness‐of‐fit values in the range $0.999999<r^{2}<1$. The corresponding residuals are shown in Supporting Information S1; Figure S1D,E, and the numerical stability of the cavity fit is confirmed in Supporting Information S1; Figure S1F.

In practice, reaction rates are often obtained by fitting the linearized form of the differential extinction, $\ln\left[{\text{Ext}}_{\infty }-\Delta \text{Ext}\left(t\right)\right]$. Because this linearization requires a value for ${\text{Ext}}_{\infty }$, the extinction at the end of the measurement is commonly used, meaning that ${\text{Ext}}_{\infty }$ is no longer treated as a fitting parameter. As shown in Figure 1D for the cell and Figure 1E and Supporting Information S1; Figure S1I for the cavity, this procedure can introduce substantial deviations from linearity if the experiment is terminated too early. Nevertheless, to extract a rate constant, the fit is typically restricted to the early time portion where $\ln\left[{\text{Ext}}_{\infty }-\Delta \text{Ext}\left(t\right)\right]$ still appears linear. However, as demonstrated in Figure 1F, the length of the selected fitting interval has a strong influence on the extracted rate constant and consistently leads to an overestimation. Note that ${\text{Ext}}_{\infty }$ differs between the cell and the cavity, as illustrated in Figure 1C, and, for the cavity, also depends on whether the monitored wavelength lies at a fringe maximum or minimum. However, this variation does not affect the extracted rate constant, as treating ${\text{Ext}}_{\infty }$ as a free fitting parameter yields identical rates in all cases.

### 3.2 Cavity Contraction

Reliable analysis of (pseudo‐)first‐order kinetics is straightforward in an ideal Fabry–Pérot cavity in which the mirror separation remains constant throughout the measurement. In practice, however, this condition is rarely met in liquid‐phase VSC experiments. Most studies to date have employed microfluidic Fabry–Pérot cavities—essentially modified spectroscopy cells or their derivatives [7, 8, 10, 11, 12, 14, 24] (see also the tutorial video from the Ebbesen lab at https://seafile.unistra.fr/d/7bb78e5a4607424f94b5/). Although more advanced designs have been proposed [17, 40, 41, 42], including devices with fixed cavity width, conventional microfluidic architectures remain the most widely used. Such cavities can exhibit several types of nonideal behavior. One is the spatial nonuniformity of the cavity width, recently characterized in detail by Michon and Simpkins [35]. In our own experiments, we observed a second type of deviation: a gradual contraction of the cavity during reaction monitoring.

**FIGURE 2:**
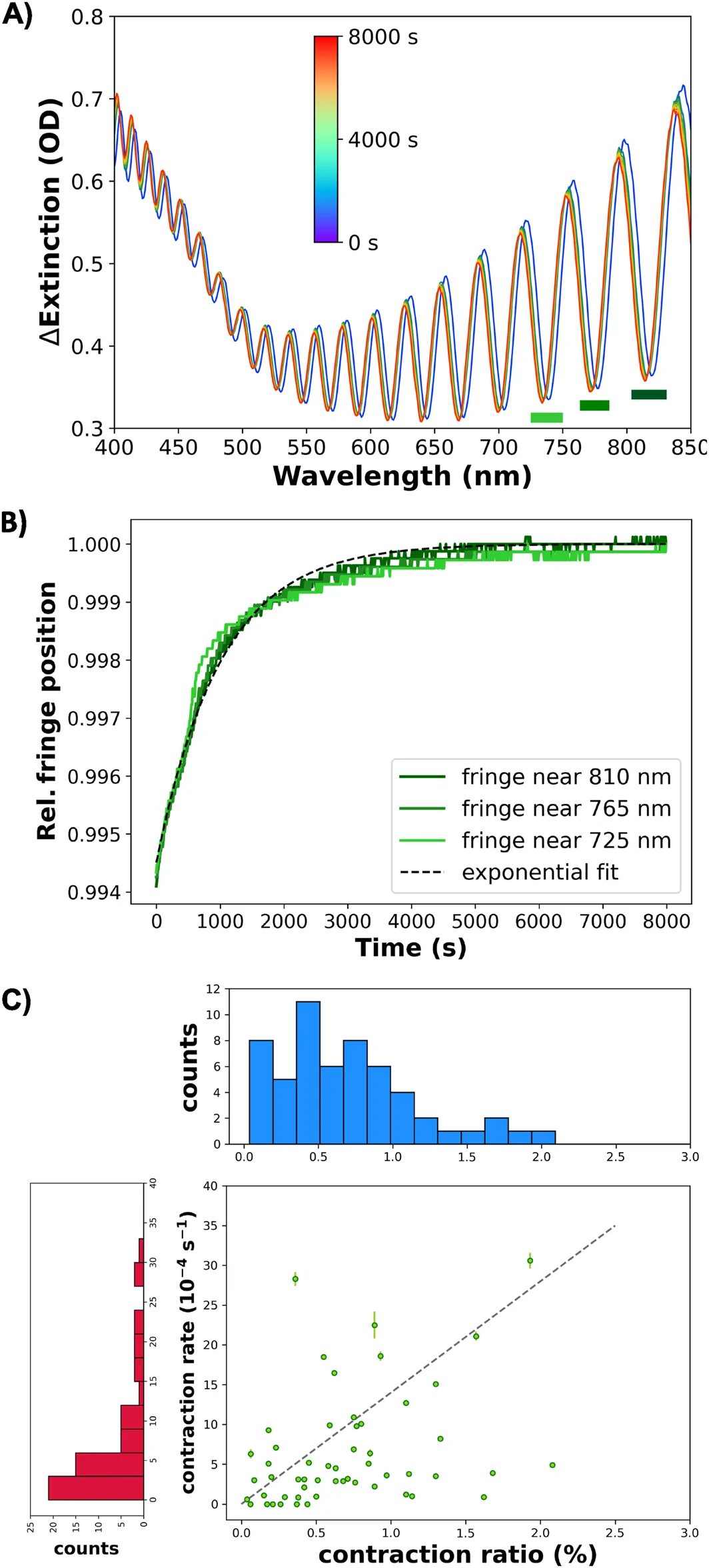
Cavity contraction during VSC chemistry experiments. (A) Example of a VSC Fabry–Pérot cavity undergoing cavity contraction after the injection of a reaction mixture with PNPA and TABF in methanol. (B) Interpolated positions of the three lower energy (longest wavelength) fringes as a function of time. The rate constant of the exponential fit (black dashed line) is $9.9\times 10^{-4}s^{-1}$. (C) Correlation plot between contraction rate and contraction ratio. The dashed line denotes the plot's diagonal. Vertical green bars denote the fitting error of the contraction rate (which for many measurements is too small to be visible on this plot).

Figure 2A shows representative unprocessed extinction spectra recorded every 1000 s from a VSC cavity following the injection of a reaction mixture containing PNPA and TBAF in methanol. The position of the fringes in these spectra is determined by the residual transmission‐mode structure of the cavity. As evident from Figure 2A, the spectra do not overlap: The fringes shift systematically toward shorter wavelengths (higher energies) over time (compare, e.g., the blue spectrum at t=0 s with the red spectrum at t=8000 s). This drift is also visible in the zoomed regions shown in Supporting Information S1; Figure S2A,B (400–440 nm and 750–850 nm, respectively). In a Fabry–Pérot cavity, the resonance frequencies of the transmission modes vary inversely with the (effective) cavity width. The observed blue‐shift therefore indicates a gradual reduction of the cavity width during the measurement. A change in the refractive index of the medium can be ruled out: The reaction converts only the solute PNPA (20 mM) without affecting the solvent methanol (∼30 M), and the fringe shifts do not correlate with the increasing absorption of the reaction product PNP in the 400–450 nm region. Importantly, this behavior is not unique to this particular cavity. We observed cavity contraction to varying degrees in every cavity sample we measured (see Supporting Information S1; Figure S3 for 15 additional examples).

The widespread occurrence of cavity contraction can be traced to the way the reaction mixture is introduced into the cavity. Before the monitoring begins, the solution must be pushed through the microfluidic Fabry–Férot cell. Under laminar flow conditions, for pressure‐driven flow in a rectangular channel, the pressure drop scales as $l^{-3}$ with the smallest channel dimension [43]. For microfluidic VSC cavities with spacer thicknesses of only a few micrometers, this scaling implies extremely large pressure drops (on the order of $10^{14}$ Pa) across the channel length. Such pressures cause the cavity assembly to deform elastically and expand slightly. Once the flow stops, the cavity relaxes toward its original geometry, that is, it contracts over time [44]. Previous UV/Vis experiments in VSC chemistry typically employed large slit widths, resulting in limited spectral resolution. This smoothing of the spectral fringes masks the visibility of the fringe drift but does not eliminate the underlying cavity contraction.

In the presence of cavity contraction, changes in extinction in the 400–450 nm region no longer reflect only the increasing PNP concentration; they also include contributions from the time‐dependent shift of the cavity fringes. It is therefore necessary to model this behavior to validate the extraction of (pseudo)first‐order kinetics under such nonideal conditions using TMM simulations.

To obtain a phenomenological description of the contraction, we extracted the relative positions of the three lowest‐energy fringes over time for each cavity measurement, normalizing the final position to 1, that is, $\delta \nu_{\text{fringe}}\left(t\right)=\left[\nu_{\text{fringe}}\left(t\right)-\nu_{\text{fringe}}\left(t=8000\;s\right)\right]/\nu_{\text{fringe}}\left(t=8000\;s\right)$. Each resulting time trace was then fitted with an exponential function to quantify the contraction rate, whereas the contraction ratio was defined as the relative change in fringe frequency between t=0 and t=8000 s. The results for the spectra in Figure 2A are shown in Figure 2B: This cavity undergoes a total relative contraction of approximately 0.6%, and its temporal evolution is well captured by a single exponential relaxation with a rate of $9.9\times 10^{-4}\;s^{-1}$.

This behavior is representative of the majority of the cavities we measured (57 in total; 15 additional examples appear in Supporting Information S1; Figure S3), although the contraction ratios and rates vary from sample to sample. A few cavities exhibit more irregular fringe shifts (e.g., Supporting Information S1; Figure S3E,I), and one cavity (Supporting Information S1; Figure S3D) requires two exponentials for distinct time windows. Nevertheless, single‐exponential relaxation provides a generally useful description for characterizing contraction in TMM simulations and guiding the relevant parameter range. Figure 2C summarizes the analysis for all measured cavities, showing histograms of contraction ratios and rates, along with a correlation plot between the two quantities. Although both parameters span substantial ranges, their correlation is negligible, as indicated by a Pearson coefficient of 0.13.

Having established both a model and quantitative measures of cavity contraction, we next simulate a (pseudo‐)first‐order reaction occurring in a homogeneous Fabry–Pérot cavity whose width relaxes exponentially over time. In this framework, the cavity is defined by a single width parameter that changes continuously according to the measured relaxation behavior. Practically, this corresponds to performing a series of TMM simulations of a Fabry–Pérot structure (as described above), each at a different time point with two time‐dependent inputs: (*i*) the dielectric function of the medium layer, which incorporates the exponentially increasing absorption at 400 nm due to the reaction, and (*ii*) the cavity width, which models the simultaneous contraction (see Supporting Information S1; Figure S4).

We begin by modeling the UV/Vis spectra of cavities contracting from different initial widths while maintaining a fixed contraction rate of $9\times 10^{-4}\;s^{-1}$. The resulting reaction time traces (i.e., the differential extinction at 410 nm) are shown in Figure 3A. It is immediately apparent that, if left uncorrected, cavity contraction has a dramatic effect on the apparent changes in the extinction at 410 nm. ΔExt now exhibits a biphasic evolution: An initial decrease (which can even lead to negative values at higher contraction levels), followed at later times by a return to exponential growth. Consequently, fitting the early portion of these traces invariably yields severely erroneous results, including negative reaction rates, for both direct exponential fitting and linearized analysis (see Figure 3 and Supporting Information S1; Figure S6). Fitting only the late‐time segments of the traces (4000–8000 s; fits with $0.999<r^{2}<1$; see residuals in Supporting Information S1; Figure S6) produces more stable estimates, but these systematically underestimate the true reaction rate as the relative contraction increases (Figure 3C).

**FIGURE 3:**
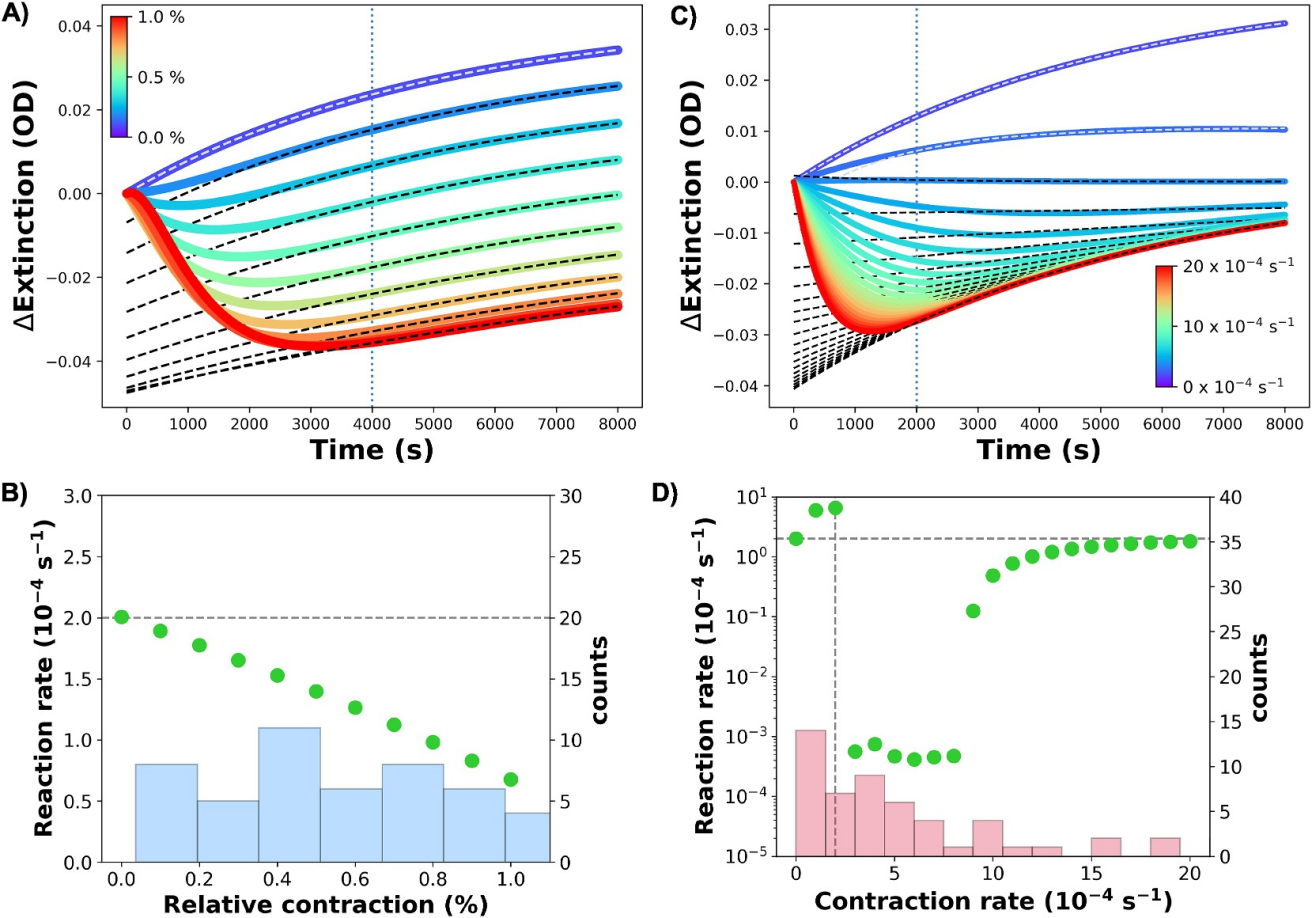
Effect of cavity contraction on reaction rate determination. (A) Reaction time traces (relative extinction at 410 nm) for cavities undergoing different degrees of contraction (colored curves). The contraction rate is fixed at $9\times 10^{-4}s^{-1}$, consistent with experimental observations. Dashed lines show exponential fits to the tail region of each trace (4000–8000 s, indicated by the vertical line). (B) Extracted rate constants (green dots) as a function of increasing relative cavity contraction $\left(\delta \nu_{\text{fringe}}\right)$. The input reaction rate of $2\times 10^{-4}s^{-1}$ is indicated by the horizontal dashed line. A histogram of experimentally observed relative contractions is shown in light blue. (C) Reaction time traces for cavities contracting at different rates (colored curves), with the total contraction fixed to 0.5%. Dashed lines show exponential fits to the 2000–8000 s window, again indicated by a vertical line. (D) Extracted rate constants (green dots) as a function of the increasing contraction rate. The input reaction rate of $2\times 10^{-4}s^{-1}$ is indicated by the horizontal dashed line. A histogram of experimentally observed contraction rates is shown in light red. Simulation parameters: Spatially uniform Fabry–Pérot cavity exhibiting an exponential decrease in the cavity width toward a limiting value of 5 μm. The medium layer displays an exponentially increasing absorption with $k_{\text{reaction}}=2\times 10^{-4}s^{-1}$. Monitoring wavelength: 410 nm.

The effects of increasing contraction rates on the reaction rate estimation are more complex and potentially more dramatic (Figure 3C and 3D). At very low contraction rates, the extracted reaction rates can be overestimated by up to a factor of three. At moderate contraction rates (2‐$8\times 10^{-4}\;s^{-1}$), the apparent reaction rates are instead underestimated—by as much as three orders of magnitude—because the cavity fringe shifts suppress the extinction increase at 410 nm. However, when the contraction rate significantly exceeds the reaction rate, contraction has little to no influence on the extracted rate constants. In this regime, the cavity width has already reached its equilibrium value prior to the kinetic measurement, such that the UV/Vis signal directly reflects the evolution of product concentration. As a result, the reaction rate can be accurately determined. As indicated by the histogram in Figure 3D, such high contraction rates are indeed observed experimentally.

### 3.3 Mitigating Cavity Contraction Artifacts

Given the substantial impact of cavity contraction and the resulting fringe shifts on the reaction rate estimation, we next examine whether standard data‐processing techniques can mitigate these artifacts. We consider three commonly used approaches: second‐order Savitzky–Golay filtering, rolling average filtering, and spectral integration. An alternative strategy would be to increase the bandwidth of the spectrometer's probe beam by widening the diffraction‐grating slit [16, 20]. Although this approach effectively smooths cavity fringes without post‐processing, it eliminates a key diagnostic of Fabry–Pérot stability and therefore removes an important indicator of cavity deformation. Moreover, as noted by Michon and Simpkins, spectral smoothing prior to conversion from transmission to extinction can introduce artificial peak shifts [35]. For these reasons, post‐processing approaches provide a more controlled and transparent means of handling cavity fringes and their temporal drift.

To illustrate the potential of these mitigation strategies, we consider a cavity undergoing a 1% contraction at the rate of $9\times 10^{-4}\;s^{-1}$, an extreme case shown in Figure 2A. Applying each of the three smoothing techniques to the simulated spectra prior to ΔExt extraction yields the red, orange, and blue curves in Figure 4A. These processed traces differ markedly from the unsmoothed black trace: Most of the distortion in the first 3000 s is suppressed, although the early portion still deviates slightly from ideal exponential behavior. Exponential fitting of the 2000–8000 s interval gives the extracted rates of

- $2.3745\times 10^{-4}\;s^{-1}$ (18 % rel. error) for Savitzky–Golay filtering
- $2.2067\times 10^{-4}\;s^{-1}$ (10 % rel. error) for rolling‐average filtering
- $2.4458\times 10^{-4}\;s^{-1}$ (22 % rel. error) for spectral integration.

**FIGURE 4:**
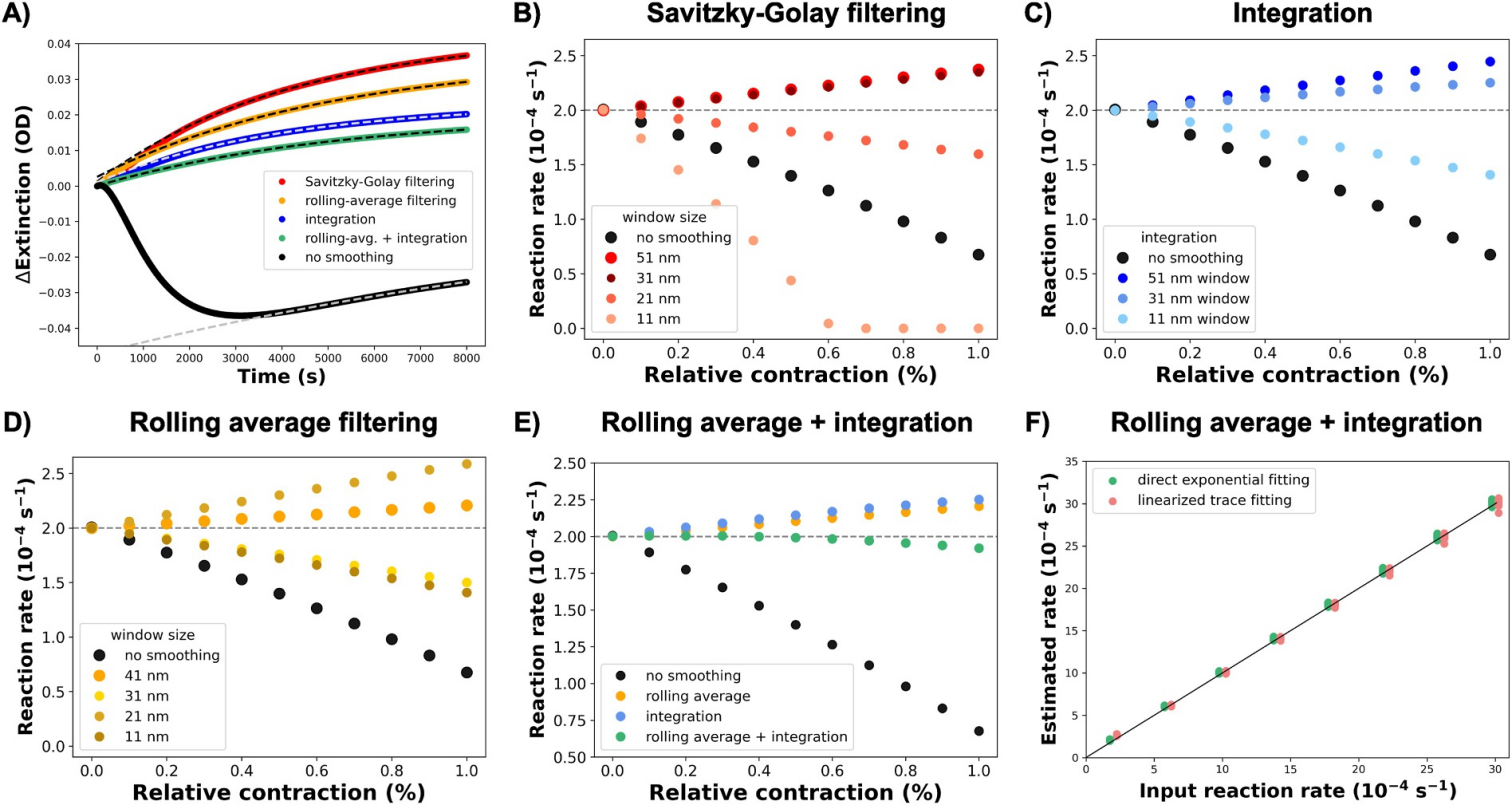
Mitigation of cavity contraction effects by data smoothing and spectral integration. (A) Reaction time traces of a cavity undergoing a 1% contraction at a rate of $9\times 10^{-4}\;s^{-1}$, extracted after applying no filtering (black curve), Savitzky–Golay filtering (red; second order, 51 nm window), rolling‐average filtering (orange; 41 nm window), or spectral integration (blue; 400–450 nm window). (B–D) Estimated reaction rates for cavities exhibiting different total relative contractions (0.0%–1.0%) while keeping the contraction rate fixed, using (B) Savitzky–Golay filtering, (C) spectral integration, and (D) rolling‐average filtering, each with multiple window sizes. (E) Estimated reaction rates for cavities with different total relative contractions (0.0%–1.0%), with the contraction rate fixed, when applying a combined procedure of rolling‐average filtering followed by spectral integration, compared to each technique alone or no smoothing. (F) Correlation between input and estimated reaction rates for the combined (rolling‐average + integration) approach. Each point corresponds to a simulation with a specific set of contraction parameters (relative contraction and rate). Simulation parameters: Spatially uniform Fabry–Pérot cavity exhibiting an exponential decrease in the cavity width toward a limiting value of 5 μm. The medium layer displays an exponentially increasing absorption with a rate constant $k_{\text{reaction}}=2\times 10^{-4}\;s^{-1}$. Monitoring wavelength: 410 nm. Standard fitting window: 2000–8000 s (individual techniques); 0–8000 s (combined approach).

All three represent a substantial improvement compared to the unsmoothed data, which yield an apparent rate of $0.6768\times 10^{-4}\;s^{-1}$, corresponding to a 68% relative error. Looking more closely at each technique individually, we can summarize the following behavior when testing against increasing levels of relative contraction:

- Savitzky–Golay filtering: A window of 21 nm (21 data points) is required to produce noticeable improvement, and even under high contraction levels, the relative error does not fall below ∼16% (Figure 4B).
- Spectral integration: Integrating over a 31 nm spectral region (31 data points) yields the best performance, with relative errors up to ∼23% and no further improvement for larger windows (Figure 4C).
- Rolling‐average filtering: The largest tested window size, 41 nm, produces the smallest relative error (Figure 4D). For the limiting case of a 1% contraction, the relative error is approximately 10%, the lowest among the three methods.

When increasing the contraction *rate* instead of the contraction *magnitude*, similar trends are observed. For all three techniques, the estimation errors are the largest at moderate contraction rates (2‐$8\times 10^{-4}\;s^{-1}$) and converge to zero as the contraction rate becomes higher.

Opting for linearized trace fitting instead of direct exponential fitting does not change the overall picture, provided that the linearized traces are computed using fitted ${\text{Ext}}_{\infty }$ values (see Supporting Information S1; Figures S7 and S8). Errors arising from cavity fringe shifts can occur alongside those introduced by an incorrect choice of ${\text{Ext}}_{\infty }$ and/or an inappropriate fitting window (see Supporting Information S1; Figures S9 and S10). This remains true even when smoothing techniques are applied, as these methods cannot fully correct distortions in the early portion of the reaction progress curve—precisely the region typically used for linearized kinetic analysis. As a result, the extracted reaction rates can be overestimated by several folds, as illustrated for Savitzky–Golay filtering and spectral integration in Supporting Information S1; Figures S9 and S10. These findings underscore once again the critical importance of using an accurate ${\text{Ext}}_{\infty }$ when performing linearized kinetic trace analysis.

Next, we ask whether the performance of the best individual method—rolling‐average filtering—can be further improved by combining it with spectral integration. Because rolling‐average filtering requires selecting a single wavelength, adding an integration step may provide additional robustness. As shown in Figure 4E, this is indeed observed: The combined approach reduces the reaction‐rate estimation error to ∼4%, whereas applying no smoothing or using either technique alone results in relative errors of 68%, 10.4%, and 12.5%, respectively. When varying the contraction rate, the combined method retrieves reaction rates with a relative error of 2.2%, compared to −6.9% and 8.2 % for rolling‐average filtering or integration alone (Supporting Information S1; Figure S11).

As a final test, we assessed the reliability of the combined rolling‐average *plus* integration approach by computing a calibration curve and verifying its linear response to a change in the input reaction rate. To this end, we simulated and analyzed reaction‐progress traces for cavities exhibiting every combination of a set of relative contractions ={0.1%,0.6%,1.0%} and contraction rates $=\left\{1,\;5,\;8,\;14,\;20\times 10^{-4}\;s^{-1}\right\}$, across a range of different input reaction rates from 2 to 30 $\times 10^{-4}\;s^{-1}$. This procedure mimics a series of polaritonic catalysis measurements in which the intrinsic reaction rate varies while each cavity exhibits a different degree of contraction. The results, shown in Figure 4F, confirm an approximately linear relationship between the input and extracted reaction rates, both when fitting the reaction time trace, ΔExt(t) directly (green circles) and when fitting the linearized trace (orange circles). The relative spread in the extracted values remains similar across the range of input rates (inset Supporting Information S1; Figure S11G). Together, these results demonstrate that the combined approach of rolling average filtering and spectral integration is the most accurate and robust method among those tested here for extracting reaction rates from contraction‐affected cavity measurements.

### 3.4 Cavity Width Inhomogeneity

In addition to cavity‐width contraction, VSC Fabry–Pérot cavities can also suffer from cavity width inhomogeneity. Such nonuniformity may arise from mirror misalignment or from bending of the mirrors under pressure when the cavity is assembled and clamped tightly. Michon and Simpkins [35] have shown that spectral distortions caused by spatial nonuniformity can lead to an altered effective molar extinction coefficient within the cavity. They further demonstrated that this static inhomogeneity alone can produce the erroneous appearance of modified reaction rates, although their analysis did not include the effects of dynamic contraction.

To examine the combined influence of both factors on apparent reaction rates, we adopt the approach of Michon and Simpkins and average a set of TMM‐calculated transmission spectra over a distribution of cavity widths centered around 5 μm with $L_{FWHM}$ = 0–110 nm. For each width in the distribution, identical contraction parameters are applied so that the instantaneous distribution of cavity widths remains consistent at all times during the simulated measurement.

The simulations described above produce UV/Vis spectra of the type shown in Figure 5 (expanded in Supporting Information S1; Figure S13A), where increasing the FWHM of the cavity‐width distribution leads to a progressive fading of the cavity fringes. This behavior is characteristic of cavity‐width inhomogeneity and corresponds both to what we have directly observed during the preparation of VSC cavities and to what Michon and Simpkins previously reported [35]. Notably, an $L_{FWHM}$ of 110 nm—only 2.2% of the nominal 5 μm cavity width—is already sufficient to attenuate the fringes almost completely. This highlights the pronounced sensitivity of Fabry–Pérot cavities to even small variations in mirror spacing.

**FIGURE 5:**
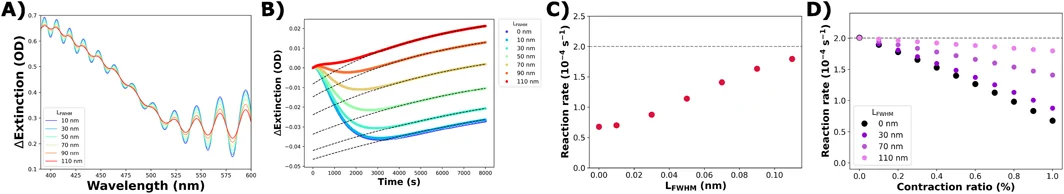
Effect of cavity width inhomogeneity. (A) Example of simulated spectra at *t* = 0 s for 6 different levels of Gaussian width broadening (as characterized by $L_{FWHM}$). (B) Reaction time traces for the formation of a PNP‐like product for 6 contracting cavities with increasing levels of Gaussian cavity width inhomogeneity. Dashed lines denote exponential curves fitted to the long‐time tails of the curves. Relative contraction = 1.0% and contraction rate = $9\times 10^{-4}\;s^{-1}$. (C) Reaction rates as obtained from direct exponential fitting to the time traces displayed in panel (B). (D) Effect of increasing cavity width inhomogeneity on the reaction rate determination in cavities experiencing different levels of contraction after injection. Contraction rate = $9\times 10^{-4}\;s^{-1}$.

Examining the complete set of simulated reaction time traces shows that increasing $L_{FWHM}$ reduces the impact of cavity contraction at early time points (Figure 5D) and also decreases the deviation of the extracted reaction rate from the true input value (Figure 5C). Systematic modeling of reactions in inhomogeneous cavities with different contraction magnitudes makes this trend even clearer: At large $L_{FWHM}$ values, the error in the fitted reaction rate grows much more slowly than in cavities with small $L_{FWHM}$, or in the homogeneous case without width broadening ($L_{FWHM}$ = 0 nm; Figure 3). Varying the contraction rate instead of the contraction ratio yields a similar behavior (Supporting Information S1; Figure S13C,D). For the model reaction considered here, cavity‐width inhomogeneity affects the simulated spectra in a manner that can partially mask the signatures of cavity contraction, similar to spectral smoothing or increased spectrometer slit width. However, this behavior is specific to the present model system and should not be interpreted as a general cancellation of artifacts. In practice, both effects remain sources of uncertainty and should be characterized, mitigated where possible, and accounted for in the rate analysis.

### 3.5 Impact of Our Findings on Previous Reports

We have used TMM simulations to identify four pitfalls that can affect the extraction of reaction rate constants in VSC chemistry experiments based on UV/Vis spectroscopy: (*i*) the choice of the limiting value ${\text{Ext}}_{\infty }$, (*ii*) the fitting interval, (*iii*) the cavity contraction, and (*iv*) cavity‐width inhomogeneity (the latter already described by Michon and Simpkins [35]). Together, these factors can introduce artifacts that affect the reliability of extracted rate constants. To assess whether the artifacts identified here could help reconcile discrepancies between four published studies reporting different reaction rates for PNPA hydrolysis in Fabry–Pérot cavities under VSC, we now evaluate to what extent these artifacts can alter the apparent rate relative to the true underlying rate.

#### 3.5.1 TenFold Rate Enhancement (Lather et al. [7])

The first report of a rate enhancement under vibrational strong coupling was published by George and coworkers [7], who observed an apparent tenfold acceleration of PNPA hydrolysis based on UV/Vis monitoring of the reaction product inside an ∼18 μm Fabry–Pérot cavity tuned to the C=O stretch vibration of both PNPA and ethyl acetate. From the methodological details provided in the paper, it appears that no spectral smoothing was applied and that the reaction progress was monitored at a single wavelength. Our analysis (Supporting Information S1; Figure S14) shows that, within the parameter ranges we have explored, an apparent reaction acceleration of this magnitude can indeed arise when the cavity contraction occurs on a similar timescale as the reaction and is large in relative terms (exceeding ∼ 0.8%). In our own experiments, we have encountered contractions of this magnitude and rate and therefore consider it plausible that the rate enhancement reported by Lather et al. [7] may be attributable, at least in part, to cavity‐contraction effects.

#### 3.5.2 Three‐Fold Rate Enhancement (Singh et al. [8])

In a follow‐up study, George and coworkers [8] revised their earlier claim of a tenfold enhancement and instead reported a threefold increase in the hydrolysis rate of PNPA, as well as a similar effect for bis‐(2,4‐dinitrophenyl) oxalate. Crucially, in this work, they applied Savitzky–Golay smoothing to the measured spectra. Our analysis (Supporting Information S1; Figure S15) shows that this processing step can substantially reduce the impact of contraction‐induced distortions on the extracted kinetic traces. Only at very low reaction rates $\left(\times 10^{-3}\;s^{-1}\right)$ could contraction‐induced artifacts lead to a modest overestimation of the extracted rate—an effect far too small to account for the reported threefold enhancement. Thus, the observations in ref. [8] cannot be explained by the contraction artifacts identified here. However, because no details were provided on how ${\text{Ext}}_{\infty }$ was determined, we cannot rule out the possibility that part or all of the reported enhancement arises from the use of an incorrect ${\text{Ext}}_{\infty }$ value.

#### 3.5.3 No Rate Modification (Wiesehan and Xiong [24])

The study by Weisehan and Xiong [24] was the first independent attempt to replicate the findings of Lather et al. [7] and provides a commendably detailed account of both the experimental procedures and the data‐analysis methodology. Our analysis shows that their use of spectral integration over a broad wavelength range effectively removes any influence of cavity contraction on the extracted time traces. As a result, the reaction rates they obtain exhibit high fidelity, with relative errors within approximately 3% (Supporting Information S1; Figure S16).

#### 3.5.4 1.8‐Fold Rate Enhancement (Verdelli et al. [9])

The group of Gómez‐Rivas employed both plasmonic nanoparticle metasurfaces and microfluidic Fabry–Pérot cavities to study the PNPA cleavage under VSC [9]. We do not comment on their metasurface experiments, which were the main focus of their study. Only limited details were provided for the Fabry–Pérot cavity measurements, preventing us from confidently reproducing their experimental configuration in our simulations. In particular, because essential information on their data‐analysis procedure is missing, we do not know whether any smoothing was applied. In light of our finding that, without smoothing, unavoidable cavity contraction can dramatically inflate the apparent reaction rate relative to the true rate, we cannot rule out the possibility that the 1.8‐fold rate enhancement reported by Verdelli et al. arises from this artifact.

## 4 Conclusion

We have used a controllable TMM framework to identify the challenges and pitfalls inherent in extracting (pseudo‐)first‐order kinetics from the UV/Vis monitoring of reactions inside commonly used Fabry–Pérot VSC cavities based on modified IR flow cells. We find that reaction rates can be significantly under‐ or overestimated due to cavity‐width contraction, which perturbs the residual cavity fringes in the UV/Vis region. Such contraction reflects the mechanical relaxation of the cavity following reactant injection and appears unavoidable given the flow mechanics associated with the small dimensions of typical IR flow cells [44].

The impact of cavity contraction can, however, be mitigated to a large extent by spectral smoothing. Among the protocols tested, the most effective procedure for extracting reliable reaction traces from UV/Vis data combines rolling‐average smoothing with subsequent spectral integration. It is unfortunate that spectral filtering has not been consistently implemented in prior reports of PNPA cleavage under VSC, as this raises questions about the reliability of some of the published rate enhancements. Contraction‐induced artifacts can act together with more traditional sources of error in first‐order kinetic analysis—such as the incorrect choice of ${\text{Ext}}_{\infty }$ or inappropriate fitting windows—but cross‐validating direct and linearized fits can help diagnose these issues. Without appropriate mitigation strategies, studies using microfluidic Fabry–Pérot cavities for VSC chemistry risk yielding unreliable results.

Beyond analysis methodology, new microfluidic VSC cavity designs may offer improved mechanical stability [40], and fixed‐width cavities have already shown promise for systematic reaction screening under VSC [15, 17, 41], albeit with certain trade‐offs. Alternative platforms, such as metasurfaces, avoid the issue of cavity‐width contraction altogether [9, 45], though they introduce distinct experimental challenges. Improvements in cavity transmission in the UV/Vis regime may also be beneficial [42].

More broadly, our results—together with those of Michon and Simpkins [35]—highlight that probing reactions or material properties inside photonic structures via spectroscopy is considerably more complex than in conventional physical chemistry or materials science settings [46]. Yet, because the spectral response of photonic structures can generally be computed using well‐established techniques (TMM for planar geometries and FDTD or FEM for other nanophotonic architectures [36]), we urge the community to validate their analysis protocols against simulations before drawing conclusions from experimental data.

## Author Contributions

**Robrecht M.A. Vergauwe:** investigation, writing – original draft, visualization, writing – review and editing, software, formal analysis. **J. Jussi Toppari:** conceptualization, funding acquisition, validation, writing – review and editing, project administration, supervision, resources. **Gerrit Groenhof:** conceptualization, funding acquisition, writing – review and editing, project administration, supervision, software, resources, validation.

## Funding

R.V. acknowledges funding from the European Research Executive Agency under the European Union's Horizon TMA MSCA Postdoctoral Fellowships—European Fellowships action (Grant Agreement No 101068621). J.J.T. acknowledges funding by the Research Council of Finland (Project 363656) and the Finnish Cultural Foundation.

## Conflicts of Interest

The authors declare no conflicts of interest.

## Supporting information


Supporting Information S1

## Data Availability

The datasets generated and/or analyzed during the current study are available from the corresponding author upon reasonable request.

## References

[1] T. Fukushima , M. Itatani , and K. Murakoshi , “Reaction Kinetics With Multiorder Cavity Modes Under Vibrational Strong Coupling,” Journal of Physical Chemistry Letters 16, no. 23 (2025): 5745–5754, 10.1021/acs.jpclett.5c00264. 40457893

[2] B. S. Simpkins , A. D. Dunkelberger , and J. C. Owrutsky , “Mode‐Specific Chemistry Through Vibrational Strong Coupling (Or a Wish Come True),” Journal of Physical Chemistry C 125, no. 35 (2021): 19081–19087, 10.1021/acs.jpcc.1c05362.

[3] B. Xiang and W. Xiong , “Molecular Polaritons for Chemistry, Photonics and Quantum Technologies,” Chemical Reviews 124, no. 5 (2024): 2512–2552, 10.1021/acs.chemrev.3c00662. PMC10941193 38416701

[4] R. Bhuyan , J. Mony , O. Kotov , et al., “The Rise and Current Status of Polaritonic Photochemistry and Photophysics,” Chemical Reviews 123, no. 18 (2023): 10877–10919, 10.1021/acs.chemrev.2c00895. PMC10540218 37683254

[5] P. Törmä and W. L. Barnes , “Strong Coupling Between Surface Plasmon Polaritons and Emitters: A Review,” Reports on Progress in Physics 78, no. 1 (December 2014): 013901, 10.1088/0034-4885/78/1/013901. 25536670

[6] H. Hiura , A. Shalabney , and J. George , “Vacuum‐Field Catalysis: Accelerating Reactions by Vibrational Strong Coupling,” ChemRxiv 7234721.v4 (2019), 10.26434/chemrxiv.7234721.v4.

[7] J. Lather , P. Bhatt , A. Thomas , T. W. Ebbesen , and J. George , “Cavity Catalysis by Cooperative Vibrational Strong Coupling of Reactant and Solvent Molecules,” Angewandte Chemie International Edition 58, no. 31 (2019): 10635–10638, 10.1002/anie.201905407. PMC6771697 31189028

[8] J. Singh , J. Lather , and J. George , “Solvent Dependence on Cooperative Vibrational Strong Coupling and Cavity Catalysis,” ChemPhysChem 24, no. 11 (2023): e202300016, 10.1002/cphc.202300016. 36745043

[9] F. Verdelli , Y.‐C. Wei , K. Joseph , et al., “Polaritonic Chemistry Enabled by Non‐Local Metasurfaces,” Angewandte Chemie International Edition 63, no. 48 (2024): e202409528, 10.1002/anie.202409528. 39159334

[10] A. Thomas , L. Lethuillier‐Karl , K. Nagarajan , et al., “Tilting a Ground‐State Reactivity Landscape by Vibrational Strong Coupling,” Science 363, no. 6427 (2019): 615–619, 10.1126/science.aau7742. 30733414

[11] K. Hirai , R. Takeda , J. A. Hutchison , and H. Uji‐i , “Modulation of Prins Cyclization by Vibrational Strong Coupling,” Angewandte Chemie International Edition 59, no. 13 (2020): 5332–5335, 10.1002/anie.201915632. 31970847

[12] R. M. A. Vergauwe , A. Thomas , K. Nagarajan , et al., “Modification of Enzyme Activity by Vibrational Strong Coupling of Water,” Angewandte Chemie International Edition 58, no. 43 (2019): 15324–15328, 10.1002/anie.201908876 PMC6856831 31449707

[13] A. Sau , K. Nagarajan , B. Patrahau , et al., “Modifying Woodward , Äìhoffmann Stereoselectivity Under Vibrational Strong Coupling,” Angewandte Chemie International Edition 60, no. 11 (2021): 5712–5717, 10.1002/anie.202013465. PMC7986062 33305864

[14] W. Ahn , J. F. Triana , F. Recabal , F. Herrera , and B. S. Simpkins , “Modification of Ground‐State Chemical Reactivity via Light–Matter Coherence in Infrared Cavities,” Science 380, no. 6650 (2023): 1165–1168, 10.1126/science.ade7147. 37319215

[15] C. Muller , M. Piejko , S. Bascil , and J. Moran , “The Diels–Alder Reaction as a Mechanistic Probe for Vibrational Strong Coupling,” Angewandte Chemie International Edition 64, no. 36 (2025): e202509391, 10.1002/anie.202509391. PMC12402871 40611390

[16] Y. Pang , A. Thomas , K. Nagarajan , et al., “On the Role of Symmetry in Vibrational Strong Coupling: The Case of Charge‐Transfer Complexation,” Angewandte Chemie International Edition 59, no. 26 (2020): 10436–10440, 10.1002/anie.202002527. PMC7318350 32220038

[17] B. Patrahau , M. Piejko , R. J. Mayer , et al., “Direct Observation of Polaritonic Chemistry by Nuclear Magnetic Resonance Spectroscopy,” Angewandte Chemie International Edition 63, no. 23 (2024): e202401368, 10.1002/anie.202401368. 38584127

[18] A. Jayachandran , B. Patrahau , J. G. Ricca , et al., “A Phenomenological Symmetry Rule for Chemical Reactivity Under Vibrational Strong Coupling,” Angewandte Chemie International Edition 64, no. 35 (2025): e202503915, 10.1002/anie.202503915. PMC12377434 40552411

[19] K. Joseph , B. de Waal , S. A. H. Jansen , J. J. B. van der Tol , G. Vantomme , and E. W. Meijer , “Consequences of Vibrational Strong Coupling on Supramolecular Polymerization of Porphyrins,” Journal of the American Chemical Society 146, no. 17 (2024): 12130–12137, 10.1021/jacs.4c02267. PMC11066862 38642054

[20] K. Joseph , S. Kushida , E. Smarsly , et al., “Supramolecular Assembly of Conjugated Polymers Under Vibrational Strong Coupling,” Angewandte Chemie International Edition 60, no. 36 (2021): 19665–19670, 10.1002/anie.202105840. 34255910

[21] K. Joseph , H. Fu , J. J. B. van der Tol , et al., “Impact of Vibrational Strong Coupling on Liquid‐liquid Phase Separation in Supramolecular Polymers,” Chemical Science 16, no. 37 (2025): 17139–17147, 10.1039/D5SC04149J. PMC12394911 40896331

[22] S. Kumar , S. Biswas , U. Rashid , et al., “Extraordinary Electrical Conductance Through Amorphous Nonconducting Polymers Under Vibrational Strong Coupling,” Journal of the American Chemical Society 146, no. 28 (2024): 18999–19008, 10.1021/jacs.4c03016. 38736166

[23] P. A. Thomas and W. L. Barnes , “Selection Bias in Strong Coupling Experiments,” Journal of Physical Chemistry Letters 15, no. 6 (2024): 1708–1710, 10.1021/acs.jpclett.3c03546. PMC10875671 38356453

[24] G. D. Wiesehan and W. Xiong , “Negligible Rate Enhancement From Reported Cooperative Vibrational Strong Coupling Catalysis,” Journal of Chemical Physics 155, no. 24 (2021): 241103–241112, 10.1063/5.0077549. 34972384

[25] M. V. Imperatore , J. B. Asbury , and N. C. Giebink , “Reproducibility of Cavity‐Enhanced Chemical Reaction Rates in the Vibrational Strong Coupling Regime,” Journal of Chemical Physics 154, no. 19 (2021): 191103, 10.1063/5.0046307. 34240900

[26] C. Climent and J. Feist , “On the S_N_2 Reactions Modified in Vibrational Strong Coupling Experiments: Reaction Mechanisms and Vibrational Mode Assignments,” Physical Chemistry Chemical Physics 22, no. 4 (2020): 23545–23552, 10.1039/d0cp04154h. 33063807

[27] T. Schnappinger and M. Kowalewski , “Do Molecular Geometries Change Under Vibrational Strong Coupling?,” Journal of Physical Chemistry Letters 15, no. 30 (2024): 7700–7707, 10.1021/acs.jpclett.4c01810. PMC11299175 39041716

[28] M. Castagnola , T. S. Haugland , E. Ronca , H. Koch , and C. Schäfer , “Collective Strong Coupling Modifies Aggregation and Solvation,” Journal of Physical Chemistry Letters 15, no. 5 (2024): 1428–1434, 10.1021/acs.jpclett.3c03506. PMC10860139 38290530

[29] J. Sun and O. Vendrell , “Modification of Thermal Chemical Rates in a Cavity via Resonant Effects in the Collective Regime,” Journal of Physical Chemistry Letters 14, no. 38 (2023): 8397–8404, 10.1021/acs.jpclett.3c02199. 37708364

[30] W. Ying and P. Huo , “Resonance Theory of Vibrational Strong Coupling Enhanced Polariton Chemistry and the Role of Photonic Mode Lifetime,” Communications Materials 5, no. 1 (2024): 2662–4443, 10.1038/s43246-024-00551-y.

[31] J. A. Campos‐Gonzalez‐Angulo , Y. R. Poh , M. Du , and J. Yuen‐Zhou , “Swinging Between Shine and Shadow: Theoretical Advances on Thermally Activated Vibropolaritonic Chemistry,” Journal of Chemical Physics 158, no. 23 (2023): 230901, 10.1063/5.0143253. 37318163

[32] L.‐Y. Hsu , “Chemistry Meets Plasmon Polaritons and Cavity Photons: A Perspective From Macroscopic Quantum Electrodynamics,” Journal of Physical Chemistry Letters 16, no. 6 (2025): 1604–1619, 10.1021/acs.jpclett.4c03439. PMC11831673 39907268

[33] J. Fregoni , F. J. Garcia‐Vidal , and J. Feist , “Theoretical Challenges in Polaritonic Chemistry,” ACS Photonics 9, no. 4 (2022): 1096–1107, 10.1021/acsphotonics.1c01749. PMC9026242 35480492

[34] K. Nagarajan , A. Thomas , and T. W. Ebbesen , “Chemistry Under Vibrational Strong Coupling,” Journal of the American Chemical Society 143, no. 41 (2021): 16877–16889, 10.1021/jacs.1c07420. 34609858

[35] M. A. Michon and B. S. Simpkins , “Impact of Cavity Length Non‐Uniformity on Reaction Rate Extraction in Strong Coupling Experiments,” Journal of the American Chemical Society 146, no. 44 (2024): 30596–30606, 10.1021/jacs.4c12269. 39466594

[36] B. E. A. Saleh and M. C. Teich , Fundamentals of Photonics, 3rd ed. (Wiley‐Interscience, Inc., 2019).

[37] G. Rosenblatt , B. Simkhovich , G. Bartal , and M. Orenstein , “Nonmodal Plasmonics: Controlling the Forced Optical Response of Nanostructures,” Physical Review X 10, no. 1 (March 2020): 011071, 10.1103/PhysRevX.10.011071.

[38] J. Orosco and C. F. M. Coimbra , “On a Causal Dispersion Model for the Optical Properties of Metals,” Applied Optics 57, no. 19 (2018): 5333–5347, 10.1364/AO.57.005333. 30117825

[39] P. Virtanen , R. Gommers , T. E. Oliphant , et al., “SciPy 1.0: Fundamental Algorithms for Scientific Computing in Python,” Nature Methods 17, no. 3 (2020): 261–272, 10.1038/s41592-019-0686-2. PMC7056644 32015543

[40] H. Yamada , G. Stemo , H. Katsuki , and H. Yanagi , “Development of a Spacerless Flow‐Cell Cavity for Vibrational Polaritons,” Journal of Physical Chemistry B 126, no. 25 (2022): 4689–4696, 10.1021/acs.jpcb.2c02752. 35723438

[41] C. Muller , R. J. Mayer , M. Piejko , B. Patrahau , V. Bauer , and J. Moran , “Measuring Kinetics Under Vibrational Strong Coupling: Testing for a Change in the Nucleophilicity of Water and Alcohols,” Angewandte Chemie International Edition 63, no. 49 (2024): e202410770, 10.1002/anie.202410770. PMC11586696 39167048

[42] L. Chen , A. M. McKillop , A. P. Fidler , and M. L. Weichman , “Ultrafast Optical Modulation of Vibrational Strong Coupling in ReCl(CO)_3_(2,2‐bipyridine),” 2025, https://arxiv.org/abs/2509.11439. 10.1515/nanoph-2025-0471 PMC12717908 41426128

[43] H. Bruus , Theoretical Microfluidics (Oxford University Press, 2008).

[44] M. A. Sahin , M. Shehzad , and G. Destgeer , “Stopping Microfluidic Flow,” Small 20, no. 20 (2024): 2307956, 10.1002/smll.202307956. 38143295

[45] Z. T. Brawley , S. Pannir‐Sivajothi , J. E. Yim , Y. R. Poh , J. Yuen‐Zhou , and M. Sheldon , “Vibrational Weak and Strong Coupling Modify a Chemical Reaction via cavity‐mediated Radiative Energy Transfer,” Nature Chemistry 17, no. 3 (2025): 439–447, 10.1038/s41557-024-01723-6. 39820806

[46] S. Renken , R. Pandya , K. Georgiou , et al., “Untargeted Effects in Organic Exciton–Polariton Transient Spectroscopy: A Cautionary Tale,” Journal of Chemical Physics 155, no. 15 (2021): 154701, 10.1063/5.0063173. 34686047

