# Supplementary material for: Toward Reliable Spectroscopic Analysis of Reaction Kinetics in Polaritonic Chemistry
Source: Nanophotonics. 2026 May 27;15(11):e70143. doi: 10.1002/nap2.70143 (PMC13642548; doi:10.1002/nap2.70143)
Supplement: Supplementary file 1 [file NAP2-15-e70143-s001.pdf]

*Supporting Information*

# Toward Reliable Spectroscopic Analysis of Reaction Kinetics in Polaritonic Chemistry

Robrecht M. A. Vergauwe,*,† J. Jussi Toppari,*,‡ and Gerrit Groenhof*,†

†*Nanoscience Center and Department of Chemistry, University of Jyväskylä, Finland*

‡*Nanoscience Center and Department of Physics, University of Jyväskylä, Finland*

E-mail: rmavergauwe@proton.me; j.jussi.toppari@jyu.fi; Gerrit.x.groenhof@jyu.fi

# Methods

## Simulations

### TMM model

All simulations of all Fabry-Perot structures were done with the Transfer Matrix Method (TMM). In TMM, a Fabry-Pérot cavity (taken at a certain time point) is modeled as a stack of layers with a defined thickness and dielectric functions (dependent on photon wavelength):

1. entry medium: air/vacuum
2. Au mirror: 10 nm
3. medium layer: 5 $\mu$m (limiting value if cavity is contracting; different for simulations of Lather et al.,[1] Singh et al.[2] and Weisehan et al.[3] - see below)
4. Au mirror: 10 nm
5. exit medium: air/vacuum

10 nm is the standard tickness for Au mirrors in VSC chemistry experiments. For each layer and each interface, and for each probe wavelength, a matrix will be constructed. For each

probe wavelength, a stack of matrices will be multiplied to one another to yield a final matrix of the simulated system. Standard formalisms for the propagation and TE/TM interface matrix, like described in ref. 4,[4] were implemented in Python (Jupyter Notebook). From the final matrix, the TE and TM-polarized transmission spectra, $T_{TE}(\lambda, t)$ and $T_{TM}(\lambda, t)$, are obtained (for clarity, these are the intensity transmission coefficients). To consider unpolarized light used in commercial UV/VIS spectrophotometers typically used for VSC chemistry experiments thus far, strictly speaking, the average of these needs to be computed. However, because only normal incidence spectra are considered in this work, $T_{TE}(\lambda, t)$ equals $T_{TM}(\lambda, t)$ and either can be taken as the cavity transmission spectrum $T_{sim}(\lambda, t)$ and converted to extinction units:

$$\mathrm{Ext}(\lambda, t) = -\log_{10}\left(\mathrm{T}_{\mathrm{sim}}(\lambda, t)\right)$$

.

The dielectric function of Au was based on the published values for the complex refractive index obtained from Au thin films by Rosenblatt et al.,[5] previously using by Michon and Simpkins in their simulations of UV/VIS of VSC Fabry-Perot cavities.[6]

### Medium layer dielectric constant and reaction simulations

The dielectric function of the medium layer $\epsilon_{\mathrm{medium}}(\omega)$ consists of a Gauss-Lorentz oscillator model $\epsilon_{\mathrm{absorber}}(\omega)$ with a constant dielectric background $\epsilon_0$. This models a typical chromophore with a single broad absorption band with a approximately gaussian-like shape, like *para*-nitrophenolate in a (near-)dispersionless organic solvent with refractive index similar to methanol. The implementation of Orosco and Coimbra[7] is chosen as this is consistent with the Kramers-Kronig relations and doesn't involve (numerically expensive) convolution (which the Brendel-Bormann model would require):

$$\epsilon_{\mathrm{medium}} = \epsilon_0 + \tilde{a} \cdot \epsilon_{\mathrm{absorber}}(\omega) = \epsilon_0 + \tilde{a}\left[\frac{s_w(z_+) + s_w(z_-)}{\chi_0}\right]$$

In this construct, $\tilde{a}$ allows us to control the magnitude of $\epsilon_{\mathrm{absorber}}(\omega)$ contribution and is for most simulations kept at $1.2 \times 10^{-1}$, while $\chi_0$ is a normalization constant and $s_w(z)$ is a dimensionless shape function given by:

$$\chi_0 = -4\sqrt{\pi} D\left(\frac{-\omega}{\sqrt{2}\sigma}\right)$$

$$s_w = i\pi \cdot w(z) + e^{-z^2}\left(\ln z + \ln\frac{-\bar{z}}{|z|^2} - i\pi\right)$$

with $w(z)$ and $D(x)$ resp. the Faddeeva function and the Dawson's function and $z_\pm$ defined as:

$$z_+ = \left(\frac{\alpha(\omega) + \omega}{\sqrt{2}\sigma}\right)$$

$$z_{-} = \left( \frac{\alpha(\omega) - \omega}{\sqrt{2}\sigma} \right)$$

$$\alpha(\omega) = \sqrt{\omega^2 + i\omega\Gamma}$$

where $\omega$, $\Gamma$ and $\sigma$ are the photon frequency, the Lorentz distribution width and Gaussian distribution width. Note that in the above, signs were chosen to be consistent with the sign convention of our TMM implementation: $\tilde{n}_{\mathrm{medium}} = \sqrt{\epsilon_{\mathrm{medium}}}$ will have $\mathrm{Im}(\tilde{n}_{\mathrm{medium}}) > 0$ and a proper orientation of the sigmoidal profile of $\mathrm{Re}(\tilde{n}_{\mathrm{medium}})$. Using this with different TMM packages may require adapting this.

In this work, we focus on simulating VSC chemistry experiments in which *para*-nitrophenolate acetate is converted to *para*-nitrophenolate for reason stated in the mean text.[1–3] To that end, we make the following choices for the parameters of the dielectric function of the medium layer, which are held fixed throughout this work:

- $\epsilon_0 = (1.30)^2$ For reference, n(MeOH) is approx. 1.31 across the visible spectrum; for most organic solvents n(Vis region) lies between 1.3 - 1.55.
- $\lambda_{peak} = 400\ nm$
- $\Gamma = 1\ cm^{-1}$
- $\sigma = 1000\ cm^{-1}$ which results in a peak FWHM of approx. 25 nm, similar to *para*-nitrophenolate.
- $\tilde{\alpha} = 1.2 \times 10^{-4}$ which results in an extinction of about 0.03-0.035 at 400 nm in both a cell and cavity geometry (*i.e.*without or with mirrors present), typical for a kinetics experiments done with short pathlengths or low concentrations of *para*-nitrophenolate.

The extinction spectra obtained with these parameters are shown in Figures 1A, S1A and S1B. The refractive index dispersion due to these absorption amounts to at most $\pm 0.0006$ RI units.

To simulate a (pseudo-)first order reaction with pro-chromophoric substance, a scaling coefficient for the amplitude parameter $\tilde{a}$ of $\epsilon_{\mathrm{GL}}(\lambda)$ with values between 0 and 1 is calculated for different time points:

$$c(t) = 1 - e^{-\mathrm{k}_{\mathrm{reaction}} \cdot t}$$

For each time point, we run a TMM simulation during which the scaling coefficient is multiplied to $\tilde{a}$ and passed to a routine for calculating $\epsilon_{\mathrm{absorber}}(\omega)$. In almost all cases, time points are chosen to run from 0 to 8000 s at 10 s intervals and spectra where computed from 390 to 850 nm with a sampling resolution of 1 nm. This matches VSC chemistry experiments we have undertaken with PNPA and TBAF. $\mathrm{k}_{\mathrm{reaction}}$ is set to $2 \times 10^{-4}$ throughout this work, save for the case of *e.g.* Figures 4F and S14 where the precision of reaction rate determination is tested for a wide range of $\mathrm{k}_{\mathrm{reaction}}$ values, and, when simulating previous works on PNPA cleavage (see Figs. S14, S15, S16).

## Reactions outside cavity

To calculate the time-dependent UV/VIS extinction spectra of a reaction taking place in standard transmission measurement cell as reference geometry, a TMM calculation is performed on an optical layer stack lacking the Au mirrors: air ($n = 1$) / medium layer / air ($n = 1$). Incorporation of the reaction and the calculation of the cell extinction spectra from the computed $\mathrm{T_{TTM}}(\lambda, \mathrm{t})$ was the same as above for the cavity.

## Cavity inhomogeneity

In order to incorporate lateral cavity width inhomogeneity, we followed the approach of Michon and Simpkins as described in.[6] Briefly, this method considers the case where a typical VSC Fabry-Perot cavities is probed over an area with a non-uniform cavity width. In practice, this manifests itself by colored Newton rings being visible when looking at/through the cavity. Michon and Simpkins illustrate this in detail and we have encountered this routinely when working with microfluidic VSC cavities. Most of the time, the Newton rings will be in cross-pattern resembling the contours of a saddle surface. In that case, there will gradients of cavity width radiating outwards from the center towards both higher and lower cavity width depending on the direction. When considering the entire probe surface as a collection of probe points for a light beam of a UV/VIS spectrometer, it is reasonable to approximate the distribution of cavity widths by a gaussian distribution characterized by a length $\mathrm{L_{FWHM}}$ proportional to the gaussian distribution standard deviation:

$$\sigma_{\mathrm{FWHM}} = \frac{L_{\mathrm{FWHM}}}{2\sqrt{2\ln 2}}$$

The transmission spectrum of a Fabry-Perot structure is taken as the weighted sum of 17 individual transmission spectra of individual cavities with different width distributed from $\mathrm{L} - 4.\mathrm{L_{FWHM}}$ to $\mathrm{L} + 4.\mathrm{L_{FWHM}}$ at $0.5.\mathrm{L_{FWHM}}$ interval with L the central cavity width and with a computed normalized weight:

$$w(L_i) = \frac{1}{\sqrt{2\pi} \cdot \sigma_{\mathrm{FWHM}}} \cdot \exp\left[\frac{-(L - L_i)^2}{2\sigma_{\mathrm{FWHM}}^2}\right]$$

We have validated the above approach for computing spectra of inhomogenously broadened cavities by comparing it with spectra computed as the sum of single-width cavity spectra, for which the cavity widths were randomly generated from a Gaussian distribution (see Fig S12). As the number of single-width spectra summed increases, the resulting spectra converge to those obtained by the Michon and Simpkins method. However, a large number of spectra, at least 1024, is needed in order to obtain acceptable convergence and noise level, which comes at the cost of a much longer compute time. Moreover, the reaction progress traces obtained still carry in them a substantial degree of noise (due to the stochastic nature of this computational process). The Michon and Simpkins method therefore seems to us the

preferable method. The reason for the appearance of noise is the stochastic nature of this type of modeling versus the deterministic nature of the Michon and Simpkins method. The contributions of lowest and highest cavity width have a very low relative weight (on the order of 0.001) but still impact the final result and thus need to be properly accounted for. This requires a number of samples on the order of 1 / 0.001 = 1000 or more. This is illustrated by Fig. S12 C, where the noise becomes more or less limited at 1024 and 4096 samples at which a more-or-less stable number of very low or very high cavity widths are incorporated at each time point.

### Data smoothing techniques and reaction time trace fitting

Three analysis techniques were tested and implemented as follows.

**Savitzky-Golay filtering** which involves fitting a polynomial to a local neighborhood of each point of the simulated spectra. Here we chose to utilize Scipy's `savgol_filter()` function with a second order polynomial and local fit windows of 51 points (values of 11, 21 and 31 points were also tested). In order to deal with the spectra limits gracefully, the first 10 data points (390-400 nm) of the simulated spectra were omitted and the keyword argument `mode='mirror'` was passed to extend the input spectrum by mirroring it. The default is `mode='interp'` and results obtained using this option are similar to using `mode='mirror'`.

**Rolling-average filtering** which involves taking a local average for each data point of a simulated spectrum. Michon and Simpkins provide a Python implementation for this filter which we use:

```
def moving_average(x_array, y_array, kernel_size = 5):
    J = kernel_size
    kernel = np.ones( 2*J+1 ) / ( 2*J+1 )
    return x_array[J:-J], np.convolve(y_array, kernel, mode='valid')
```

A smoothing window of 41 data points was standard (window sizes of 11, 21 and 31 were also tested). In the latter two cases, the reaction time trace was taken at the first points of the smoothed spectra (413 and 415 nm resp.).

**Integration** which involves integrating a local region of each simulated spectra of 11, 31 or 51 points. This corresponds to the 405-415, 395-425 and 400-450 nm spectral regions. The integration result was divided over the length of the integration window to allow easy comparison with other spectra (raw and filtered).

**Integration after rolling-average filtering**. After application of a rolling-average filter over a 41 point window (so 20 points on each side of each data point), the resulting extinction spectrum was integrated between 410 and 430 nm.

The progress of the reaction was either inferred from the differential absorption at 410 nm with respect to the absorption at time 0, *i.e.* $\Delta\text{Ext}(410\text{ nm}, \text{t}) = \text{Ext}(410\text{ nm}, \text{t}) - \text{Ext}(410\text{ nm}, 0\text{ s})$ (cavity) or from the integrated differential absorption spectrum (cell).

Direct fitting of the time traces of the differential absorption/extinction was done with

one of these two exponential model functions:

$$\Delta\text{Ext}(t) = \text{Ext}_\infty \left(1 - e^{-\text{k}_\text{fit} t}\right) \tag{1}$$

or

$$\Delta\text{Ext}(t) = \text{Ext}_0 \left[1 - e^{-(t-t_0)\text{k}_\text{fit}}\right] + \text{Ext}_\text{bck} \tag{2}$$

where $\text{k}_\text{fit}$ is the fit parameter reflecting the reaction rate; any deviation from $\text{k}_\text{reaction}$ must thus reflects an artifact. The exponential function in equation 1 is used when examining an ideal, homogeneous cavity (Figures 1 and S1), as there is no time delay and no background extinction in these simulations (typically present during experiments). When cavity contraction is included (see Figure 3, S5 and S6), fitting is performed with an 4-parameter exponential model function 2 which is essentially an exponential function free to be translated in both the dimensions of time and extinction through the parameters of $t_0$ and $\text{Ext}_\text{bck}$. This is a fit function which is typically used in analysis of experimental data as, in this case, one will typically encounter measurement time delays (stemming from the delay between mixing the reaction compounds and starting the data collection on the spectrophotometer) and background signals (*e.g.*stray light, uncorrected reflections). To be clear, our modeling assumes neither of these two phenomena to be present (so the reaction starts at t = 0 s with an initial product concentration of 0 M). We employ this fit function here because we want to characterize parts of the reaction time traces affected by contraction artifacts with a method which would normally get used in an experimental setting. The $t_0$ and $\text{Ext}_\text{bck}$ thus do not possess any physical meaning, but are necessary because the presence of artifacts in the reaction time traces. All fitting was done using the *scipy.optimize.curve_fit*() routine, which performs least-squares curve fitting based on the Levenberg-Marquardt algorithm (no fit constraints were used).[8]

It is standard practice in physical chemistry to analyze (pseudo-)first-order reaction kinetics data by converting this to a linearized form:

$$\text{Ext}(t) = \text{Ext}_\infty \left[1 - \exp\left(-\text{k}_\text{reaction} \cdot t\right)\right] \Longleftrightarrow$$
$$\ln\left[\text{Ext}(t) - \text{Ext}_\infty\right] = \ln\left[\text{Ext}_\infty \cdot \exp\left(-\text{k}_\text{reaction} \cdot t\right)\right] = \ln\left[\text{Ext}_\infty - \text{k}_\text{reaction} \cdot t\right]$$

This practice gained currency at a time when computer-aided data analysis/fitting was not common and stuck with the field ever since. The advantage of this is that:

1. verification of first order conditions is straightforward. Both first and second order kinetics data (*i.e.* curves of the order of $\exp(-t)$ and $\frac{1}{t}$) look roughly similar to the naked eye when plotted on a normal plot. After conversion to the form stated above, they don't anymore.

2. only an accurate value of $\text{Ext}_\infty$ is needed, making the need for accurate knowledge of the starting concentrations/extinctions/... unnecessary. In chemical kinetics measure-

ments, there is more than often a time delay between mixing the reagents, which is the true start of the reaction, and the start of data collection. Because reagent conversion occurs at its fastest in the beginning of the reaction, determining accurate starting values can be error-prone.

However, a downside of analysis through linearized time traces is that a precise value of $\mathrm{Ext}_\infty$ must be used. If a correct value is used, $\ln[\mathrm{Ext(t)} - \mathrm{Ext}_\infty]$ will be linear:

$$\begin{aligned} \ln[\mathrm{Ext}_\infty - \mathrm{Ext}(t)] &= \ln\{\mathrm{Ext}_\infty - \mathrm{Ext}_\infty[1 - \exp(-\mathrm{k}_{\mathrm{reaction}} \cdot t)]\} \\ &= \ln \mathrm{Ext}_\infty + \ln[-\exp(-\mathrm{k}_{\mathrm{reaction}} \cdot t)] \\ &= \ln \mathrm{Ext}_\infty - \mathrm{k}_{\mathrm{reaction}} \cdot t \end{aligned}$$

If a bad value $Ext_\infty^{bad}$ is used, the linearized plot will curve off:

$$\begin{aligned} \ln\left[\mathrm{Ext}_\infty^{bad} - \mathrm{Ext}(t)\right] &= \ln\left\{\mathrm{Ext}_\infty^{bad} - \mathrm{Ext}_\infty[1 - \exp(-\mathrm{k}_{\mathrm{reaction}} \cdot t)]\right\} \\ &= \ln \mathrm{Ext}_\infty + \ln[r - \exp(-\mathrm{k}_{\mathrm{reaction}} \cdot t)] \end{aligned}$$

with $r = \mathrm{Ext}_\infty^{bad}/\mathrm{Ext}_\infty - 1 > 0$. This is illustrated in Figure S1 panel I, where incorrect values for $\mathrm{Ext}_\infty$ are selected on purpose, and in panels C and D of Figure 1. In the latter, two guesses for $\mathrm{Ext}_\infty$ are tested against the 'true' value as determined from a simulation done with the full value of $\tilde{a} = 1.2 \times 10^{-4}$ (no scaling coefficient applied), which represents a simulate at 'infinite time' so to speak.

For most of the data in this paper the parameter $\mathrm{Ext}_\infty$ was derived from previous exponential fitting. When fitting with Eq. 1, $\mathrm{Ext}_\infty$ is just one of the two parameters. When fitting with Eq. 2, $\mathrm{Ext}_\infty = \mathrm{Ext}_\mathrm{o} + \mathrm{Ext}_{\mathrm{bck}}$.

### Simulation of previously published experiments

To simulate the UV/VIS cavity measurements published by the groups of Jino George and Wei Xiong in their 3 papers,[1–3] the following general assumptions were made:

- the same model for PNP, generated by pseudo-first order reaction from PNPA, was used as for our previous simulated (see above). Parameters of the Gaussian-Lorentzian dielectric function were kept the same: $\lambda_{peak} = 400\ nm$, $\sigma = 1000\ cm^{-1}$, $\tilde{\alpha} = 1.2 \times 10^{-4}$ and $\epsilon_0 = (1.30)^2$.

- The 100 nm $\mathrm{SiO_x}$ insulating layers were omitted for simplicity's sake as, in practice, they will not have any discernible influence on the measured UV/VIS spectra as the cavities width are approx. 18-20 $\mu$m .

- No information could be found in all 3 papers on the extent of cavity inhomogeneity present in the measured cavities. Because cavity inhomogeneity effectively acts a form of smoothing (see Figure 5 and discussion thereof in the main text), we opted to not include this in our simulations. The simulations thus represent a worst-case scenario.

- No information could be found in all 3 papers on the nature of cavity shifting. So, we only examine cavity contractions of the kind we expect from our experiments and also focus on in other parts of this paper, being contractions of 0 to 1 % (see Figure S14) or of 0 to 10 % (see Figure S15) with rates from 0 to 20 $10^{-4}$ $s^{-1}$. Our reasoning is that both groups used a flowcell broadly similar (be it commercially sourced) to what we have used.

**Parameters simulations Lather et al. Angew Chem 2019** Spectra from 370 to 600 nm (sampled at 1 nm intervals) were computed for 0 to 600 s in 1 s intervals and reaction progress traces were directly taken as the differential extinction at 407 nm without any prior smoothing of the spectra. Although the main text of the article mentions spacer width of approx. 18 $\mu$m being using, we instead relied on the table in the Supplementary Information of this paper with free spectral range figures of all the measured cavities when still empty before reaction monitoring (going from 230.5 to 251.3 $cm^{-1}$ ). From this we calculated the range of measured cavity widths ($l_{cav} = \frac{1}{2.FSR}$ with $n = 1$ for air), which starting at 19.9 $\mu$m and going to 21.7 $\mu$m . Both values correspond to measurements of OFF resonant cavities, *i.e.*cavities with no VSC of the C=O stretch vibration of ethyl acetate/PNPA. Additionally, we chose to simulate the ON resonant cavity with an FSR of 240.46 $cm^{-1}$ and a calculated cavity width of 20.86 $\mu$m . As no mention was found regarding how $Ext_\infty$ was determined, we chose to extrapolate it from exponential fitting. This represents a best case scenario and also avoided the anomalous results (*i.e.*negative fitted rates) we occasionally would encounter when choosing to take $E_\infty$ as the maximum of the simulated trace. The linearized reaction trace was fitted at the first 60 data points, which corresponds to the first 60 s of the measurement.

**Parameters simulations Singh et al. Chem Phys Chem 2023** Spectra from 370 to 600 nm (sampled at 1 nm intervals) were computed for 0 to 1000 s in 10 s intervals. Simulated spectra were smoothed with an 2th order Savitzky-Golay filter (51 nm filtering window) and the reaction progress traces are subsequently taken at 400 nm. We chose to simulated cavity widths of 18 $\mu$m and 18.7 $\mu$m . The first value is mentioned throughout the article, the second value was computed from only of the only mention of a value for an empty cavity free spectral range in the entire article. This value of 267 $cm^{-1}$ was for a cavity near resonance to the C=O stretch vibration of ethyl acetate/PNPA. As no mention was found regarding how $Ext_\infty$ was determined, we chose to obtain this as the maximum of the simulated reaction trace (no anomalous results were obtained here as Savitzky-Golay filtering suppressed almost all distortions of cavity contraction). The linearized reaction trace was fitted on the first 11 data points, which corresponds to the first 100 s of the measurement.

**Parameters simulations Wiesehan et al. J Chem Phys 2021** Spectra from 370 to 850 nm (sampled at 1 nm intervals) were computed for t = 0 to 600 s at 1.5 s intervals and for t = 600 to 3600 s at 10 s intervals. Smoothing was done by integration over the 375

to 425 nm spectral region. We chose to simulate cavity widths of 18 $\mu$m and 19.55 $\mu$m . The first value is mentioned throughout the article, the second value is mentioned in the Supplementary Information in the context of a path length calibration between a cell and cavity (otherwise no further mentions of cavity widths or of empty cavity free spectral ranges widths were noted). $Ext_\infty$ was determined as the maximum of the simulated reaction trace as mentioned in the paper. Fitting linearized reaction traces was done on a window of the first 200 points, which corresponds to the first 300 seconds of the measurement.

## Cavity contraction experiments

The experimental cavity contraction data presented in Figures 2 and S3 comes from VSC chemistry experiments with *para*-nitrophenol acetate (PNPA) and tetrabutylammonium fluoride (TBAF) in methanol (MeOH) using a home-made microfluidic VSC Fabry-Perot cavities. These VSC cavities consist out of a pair of insulated mirrors on an optically polished 3 mm thick 1 inch $BaF_2$ IR transparent windows (Crystran Ltd, UK) with is sandwiched in between a spacer cut from polypropylene film of nominally 4 $\mu$m , but in practice $4.5-5.5$ $\mu m$, thickness (SKU 1000038738 from Goodfellow Cambridge Ltd, UK) . The mirrors were manufactured by thermally evaporating a 10 nm Au layer with a 0,5 nm Ti adhesion layer ($P_{evap} = 1 - 4 \times 10^{-8}$ mbar, $v_{evap} = 0.2 - 0.3$ nm/s) after plasma cleaning (REI, Oxford instruments Ltd, UK), followed by the growth of an approximately 80 nm $Al_2O_3$ insulating layer using ALD (800 cycles at 200 C using trimethyl aluminum and water as precursors; TFS 200 from Beneq OY, Finland). Mirrors and spacer were assembled together in custom made metal holder (steel or brass, each with a rectangular observation port cut out of 18 mm by 11 mm) using 0.5 mm thick teflon liners for protection and even pressure distribution. The holder consisted out of a top and bottom part (4 and 10 mm thick respectively) assembled together with 6 M3 screws with 3 spring disks (0.5 mm thick each) equidistantly placed along the edge. Playing with tightness of the screws can result in the appearance of colored Newton rings. In many cases, a spot of a single color of at least 5 mm across can be obtained, which was subsequently selected for spectroscopic interrogation by taping a metal mask with 4 or 5 mm diameter hole to the holder. The cavity was allowed to settle overnight before measurement the next day. The free spectral range increases typically 50 to 100 $cm^{-1}$ and is due to relaxation of stresses in the various parts of the cavity assembly.

Before injection and starting the measurement, each cavity was mounted in a temperature control block mounted on a flip mount equipped with two orthogonal micrometer positioning stages for aligning the cavity to the spectrometer beam. This temperature control block was connected to a refrigerated water recirculator (Thermo Haake C/DC class C10-K10). A 2 mm wide PT100 thermocouple connected to a Keithley 2001 digital mulitmeter (4-point resistance measurement) was inserted to a 2 mm hole in the bottom metal holder for monitoring the temperature of the cavity assembly during each experiment. Before injection and measurement, the cavity was brought to a temperature between 24.9 and 25.1°C and allowed to reach thermal equilibrium during a period of at least one hour. During UV/VIS

reaction monitoring, the temperature was manually controlled, using the water recirculator, to be in between 24.9 and 25.2°C at all times.

The reaction mixture consisted out of 540 $\mu l$ of a 6 M of TBAF stock (abcr GmbH, Germany; tetra-n-butylammonium fluoride trihydrate 97%, AB112723 , CAS 87749-50-6) in MeOH (analytical grade) mixed with 60 $\mu l$ of 200 mM stock solution of PNPA (Sigma-Aldrich/Merck GmbH, Germany; 4-nitrophenol acetate 98%, N8130, CAS 830-03-5) in MeOH. Using a 1 ml Luer syringe this mixture was quickly injected into the cavity using 4 mm O.D. plastic tubing and Press-Lock fittings (KJL04-M3 from SMC) with M3 screw threads for easy attachment to threaded holes through the top holder. After injection the syringe was generally left stuck on the input port side, while the tubing on the output port was sealed with a plug of telfon tape and parafilm.

UV/VIS spectra were collected on an Agilent Cary 8454 spectrophotometer (Example shown in Fig. 1A with zooms in Figure S2). This instrument is equipped with both a halogen and deuterium lamp and illuminates the sample with single light beam before analysis with a spectrometer and detection on a CCD array. Full spectra are thus collected in a single shot. For a single experiment, a time series of 800 spectra is collected at 10 s intervals, representing a reaction time interval spanning from 0 s to 8000 s. The light integration time for each spectrum is 0.5 s. A black cloth was used to cover the VSC cavity mounted in the spectrometer and shield it from ambient light.

To characterize the contraction of each measured cavity, the 3 fringes located at the lowest photon energy of each recorded spectra, *i.e.* between 720-840 nm, were tracked. Because the inverse relationship between photon energy and wavelength, the fringes in the red spectral region can be tracked with a greater energy resolution than those in more blue-shifted regions. The cusps, *i.e.*the low extinction dips, rather the high extinction peaks were chosen because the signal-to-noise ratio near the former is usually better than for the latter. To determine the exact position (*i.e.*frequency/energy) of each fringe's cusp, the region around the cusp was selected, interpolated to a 0.1 nm resolution (using *scipy.interpolate.interp1d*( *kind=cubic* )) and fed to the *scipy.signal.find_peaks*() algorithm.[8] The time traces of the fringe frequencies, $\nu_{\mathrm{fringe}}(\mathrm{t})$, were normalized the last measured value (*i.e.*at t = 8000 s), setting the final value to 1, *i.e.*, $\delta\nu_{\mathrm{fringe}}(\mathrm{t}) = [\nu_{\mathrm{fringe}}(\mathrm{t}) - \nu_{\mathrm{fringe}}(\mathrm{t} = 8000\ \mathrm{s})]/\nu_{\mathrm{fringe}}(\mathrm{t} = 8000\ \mathrm{s})$. The trace of the cusp of the fringe with the lowest frequency/energy was then fitted with an exponential function (2) to quantify the contraction rate, while the relative contraction was defined as the relative change in fringe frequency between $t = 0$ and $t = 8000$ s).

# Additional data

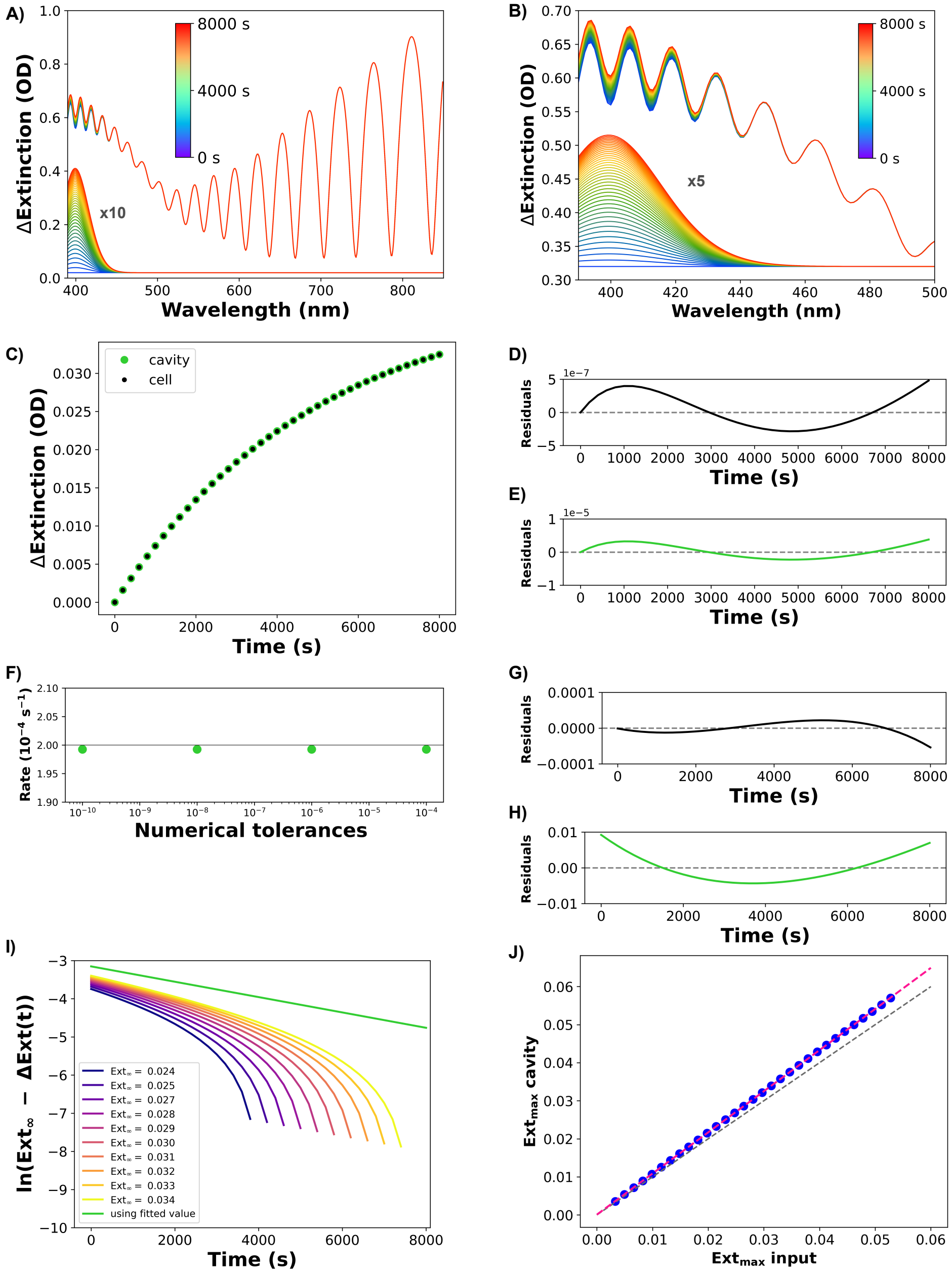


**Figure S1. First order kinetics analysis in an ideal VSC cavity: additional data. A)** Full rendered and **B)** zoomed in version of Figure 1A. Simulated UV-Vis cavity extinction spectra of an ideal Fabry-Perot VSC cavities together with the extinction spectra of the absorbing layer (multiplied resp. 10- and 5-fold for clarity). Simulations of Fabry-Perot cavity with perfectly parallel Au mirrors (10 nm) held at a constant 5 $\mu$m with a medium with exponentially increasing absorption at 400 nm over time with a rate of $k_{reaction} = 2\times10^{-4}\ s^{-1}$). **C)** Rescaled reaction progress curves for cell and cavity simulations shown in Figure 1B. **D)** and **E)** Residuals of exponential fits to reaction time traces of resp. the cell (black) and cavity (green) in Figure 1B. **F)** Numerical stability of exponential fitting cavity reaction time traces. **G)** and **H)** Residuals of the linear fits to the linearized reaction time traces calculated with an extrapolated $Ext_\infty$ value of the cell (black) and cavity (green) in resp. Figure 1C and 1D. **I)** linearized reaction time traces computed with different values for $Ext_\infty$. **J)** Relationship cell extinction and cavity extinction. Data plot as indigo circles, red and black dashed line denote resp. the linear fit and diagonal of the plot. Different cavities with different medium layer dielectric functions with varying values of a were simulated to obtain the cavity extinction at 410 nm. For each simulation, the cell extinction was computed directly from the medium layer dielectric function.

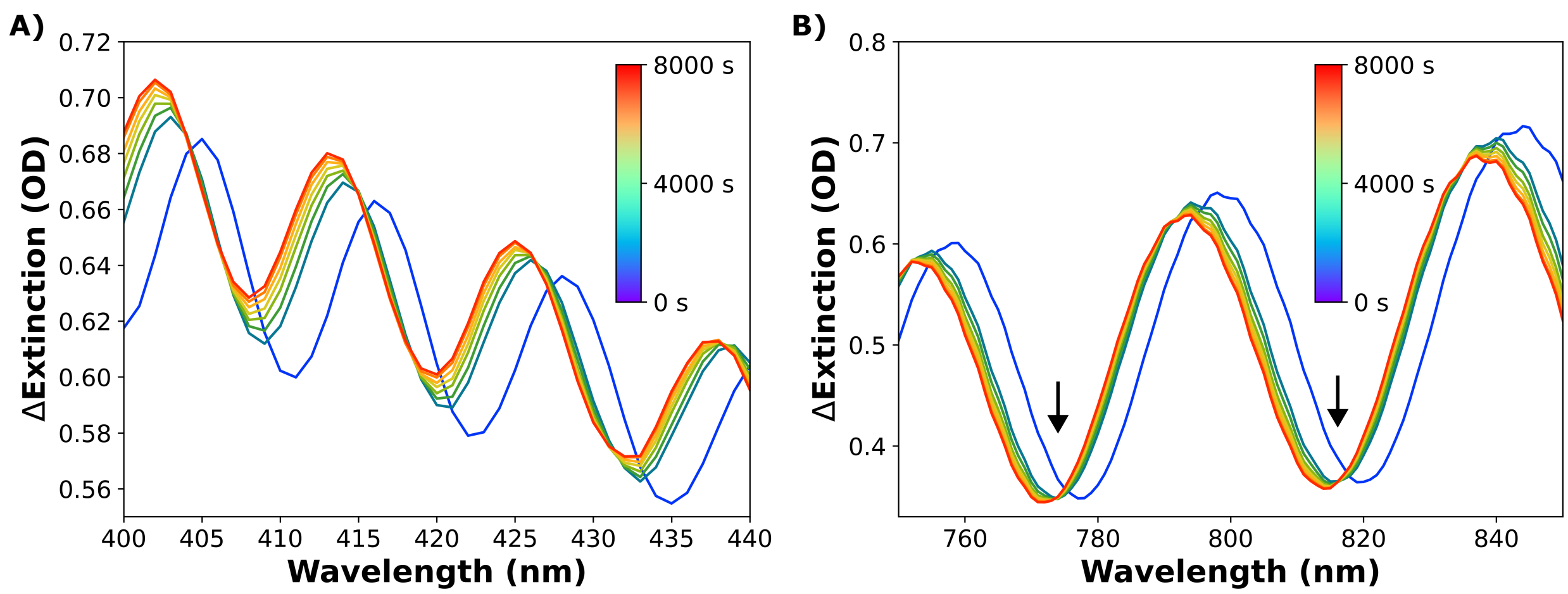


**Figure S2. Fabry-Perot cavity contraction during a VSC chemistry experiment. A)** and **B)** Zooms of the spectra displayed in Figure 2A. **A)** Zoom of the spectral region containing the absorption peak of *para*-nitrophenolate. **B)** Zoom of the spectral region containing the fringes used for tracking the contraction of the cavity over time. Displayed spectra taken from 0 s to 8000 s at 1000 s intervals.

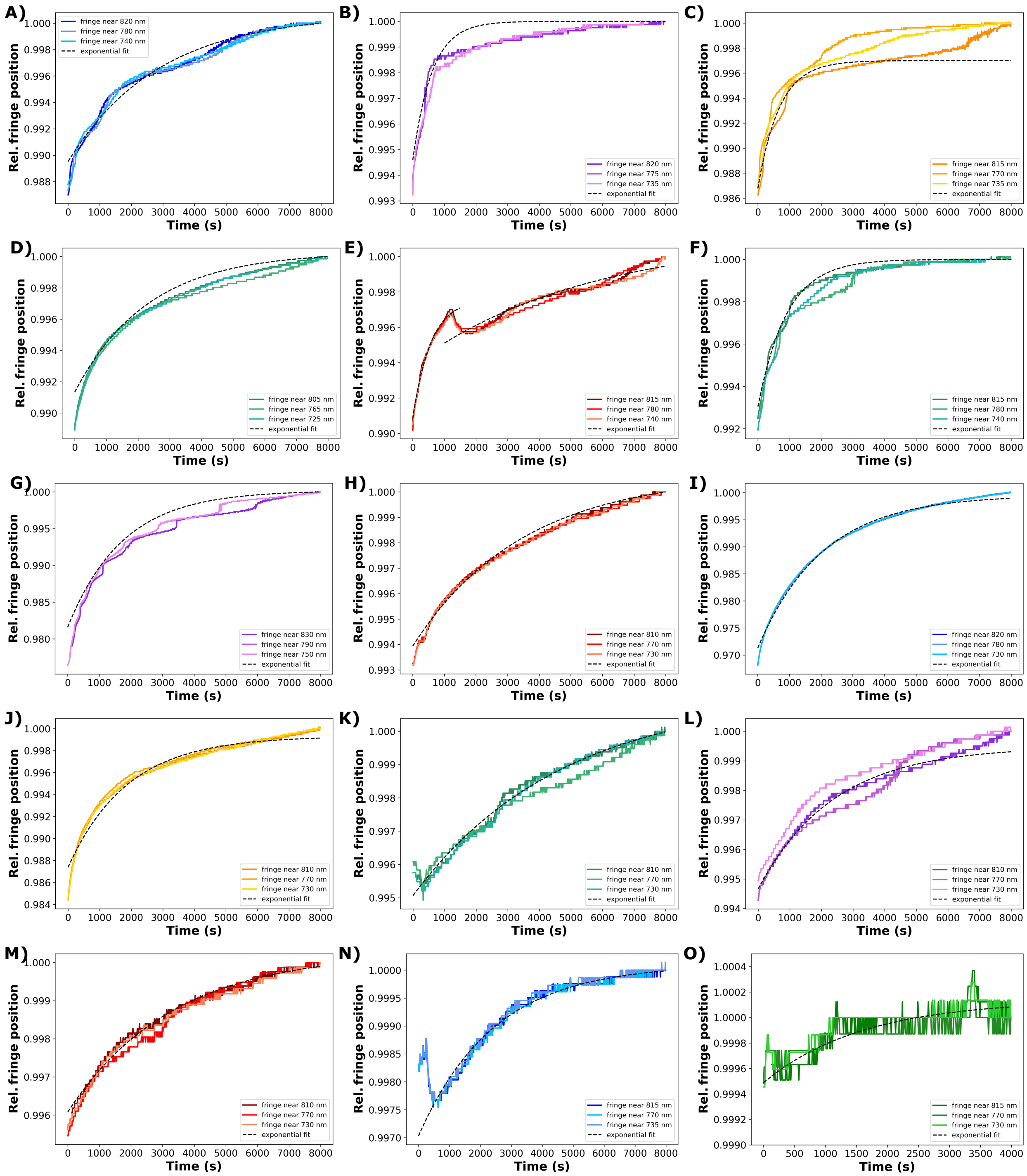


**Figure S3. Examples of cavity contraction during VSC chemistry experiments.** Time traces of relative energy (spectral position) of the dips of the three lowest energy UV/VIS fringes (see Fig. S2B). Fit of the time trace of the lowest energy cusp plotted as a dashed black line. Data shown for a sample of 15 cavities out of 57 measured and analyzed.

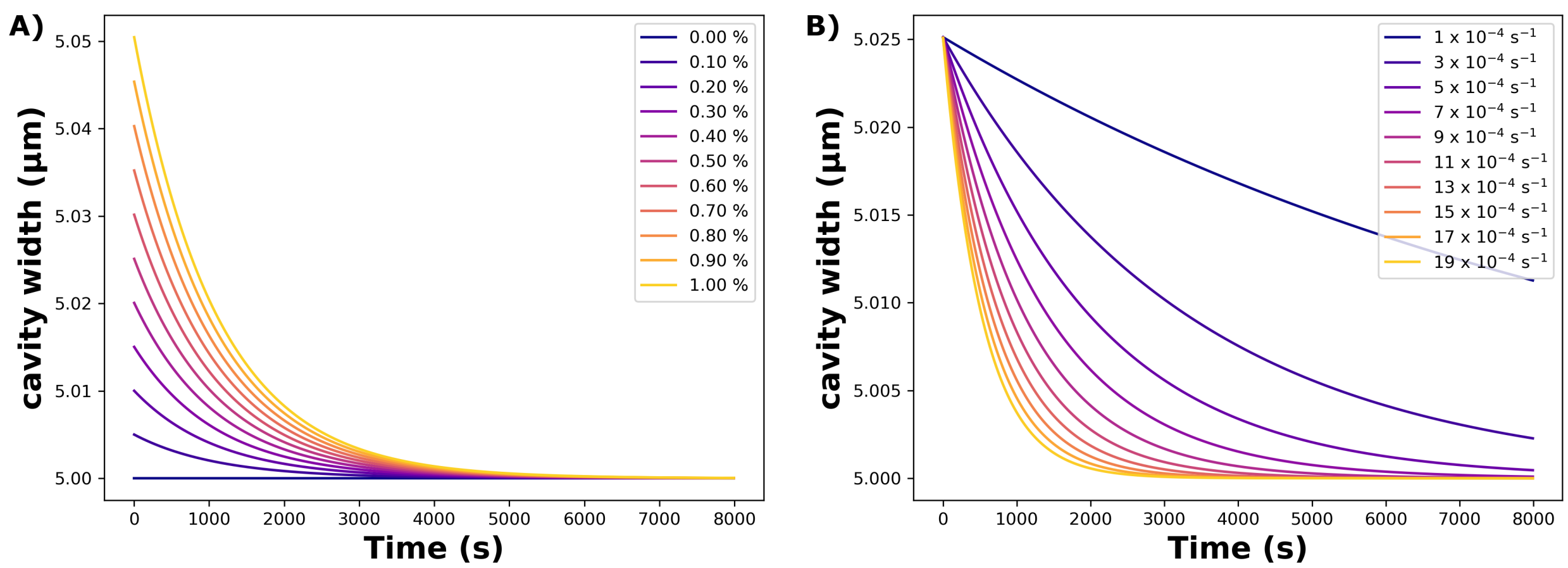


**Figure S4. Cavity widths vs time used for simulating contracting cavities.** A) Cavity widths for simulations with varying relative contractions. $k_{reaction} = 9 \times 10^{-4}$ $s^{-1}$ for all plotted cavity width traces. B) Cavity widths for simulations with varying contraction rates. Relative contraction = 0.5% for all plotted cavity width traces.

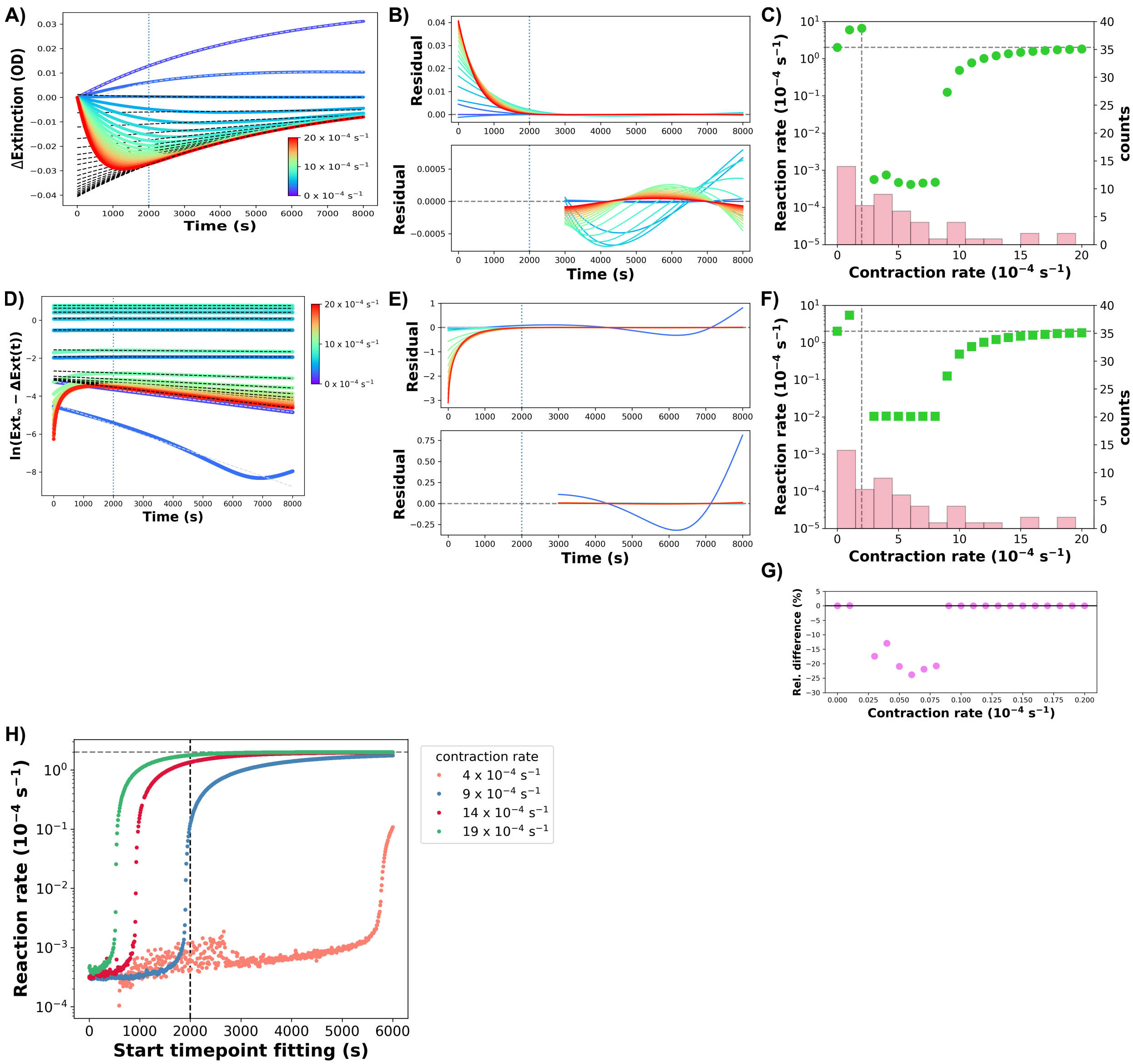


**Figure S5. Effect of contraction rate on kinetics measurements in a homogeneous, contracting cavity.** **A)** Simulated reaction time traces for $k^{cavity}_{contraction} = 0 - 20 \times 10^{-4}$ $s^{-1}$ together with exponential fits (black dashed lines). Fitting performed between 2000 and 8000 s. **B)** Residuals of the exponential fits of panel A. Upper: full simulation time window; lower: zoom on the 3000-8000 s time window. **C)** Cavity reaction rate (green circles) extracted from direct exponential fitting vs cavity contraction rate. Experimentally observed distribution of cavity contraction rates shown in transparent red (see Figure 2C). **D)** Linearized reaction time traces for $k^{cavity}_{contraction} = 0 - 20 \times 10^{-4}$ $s^{-1}$ together with linear fit curves (black dashed lines). Fitting performed between 2000 and 8000 s. **E)** Residuals of the exponential fits of panel D. Upper: full simulation time window; lower: zoom on the 3000-8000 s time window. **F)** Cavity reaction rate (green squares) extracted from linearized trace fitting vs cavity contraction rate. Experimentally observed distribution of cavity contraction rates shown in transparent red (see Figure 2C). **G)** Relative difference between reaction rates obtained by direct trace fitting (panel C) and linearized trace fitting (panel F). **H)** Influence of the starting point of the fit window on extracted reaction rate for 4 selected cavity contraction rates. Data of simulations with a constant cavity relative contraction = 0.5 % and $k_{reaction} = 2 \times 10^{-4}$ $s^{-1}$.

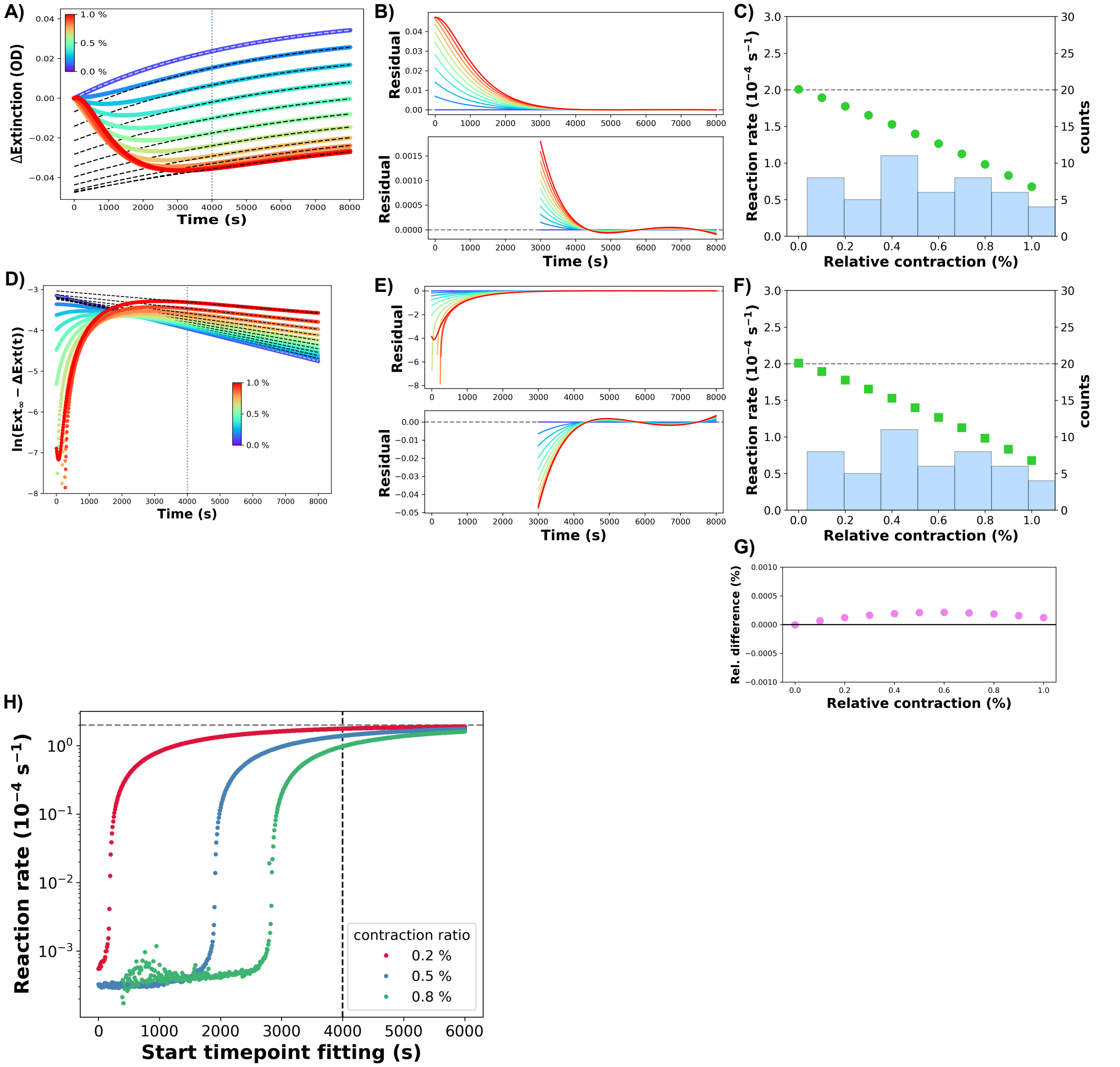


**Figure S6. Effect of relative contraction on kinetics measurements in a homogeneous, contracting cavity. A)** Simulated reaction time traces for relative contractions of 0 to 1 % together with exponential fits (black dashed lines). Fitting performed between 4000 and 8000 s. **B)** Residuals of the exponential fits of panel A. Upper: full simulation time window; lower: zoom on the 3000-8000 s time window. **C)** Cavity reaction rate (green circles) extracted from direct exponential fitting vs cavity relative contraction. Experimentally observed distribution of cavity relative contractions shown in transparent blue (see Figure 2C). **D)** Linearized reaction time traces for relative contractions of 0 to 1 % together with linear fit curves (black dashed lines). Fitting performed between 4000 and 8000 s. **E)** Residuals of the exponential fits of panel D. Upper: full simulation time window; lower: zoom on 3000-8000 s time window. **F)** Cavity reaction rate (green squares) extracted from linearized trace fitting vs cavity contraction rate. Experimentally observed distribution of cavity relative contractions shown in transparent blue (see Figure 2C). **G)** Relative difference between reaction rates obtained by direct trace fitting (panel C) and linearized trace fitting (panel F). **H)** Influence of the starting point of the fit window on extracted reaction rate for 3 selected cavity relative contractions. Data of simulations with a constant $k_{contraction}^{cavity} = 9\times10^{-4}$ s$^{-1}$ and $k_{reaction} = 2 \times 10^{-4}$ s$^{-1}$.

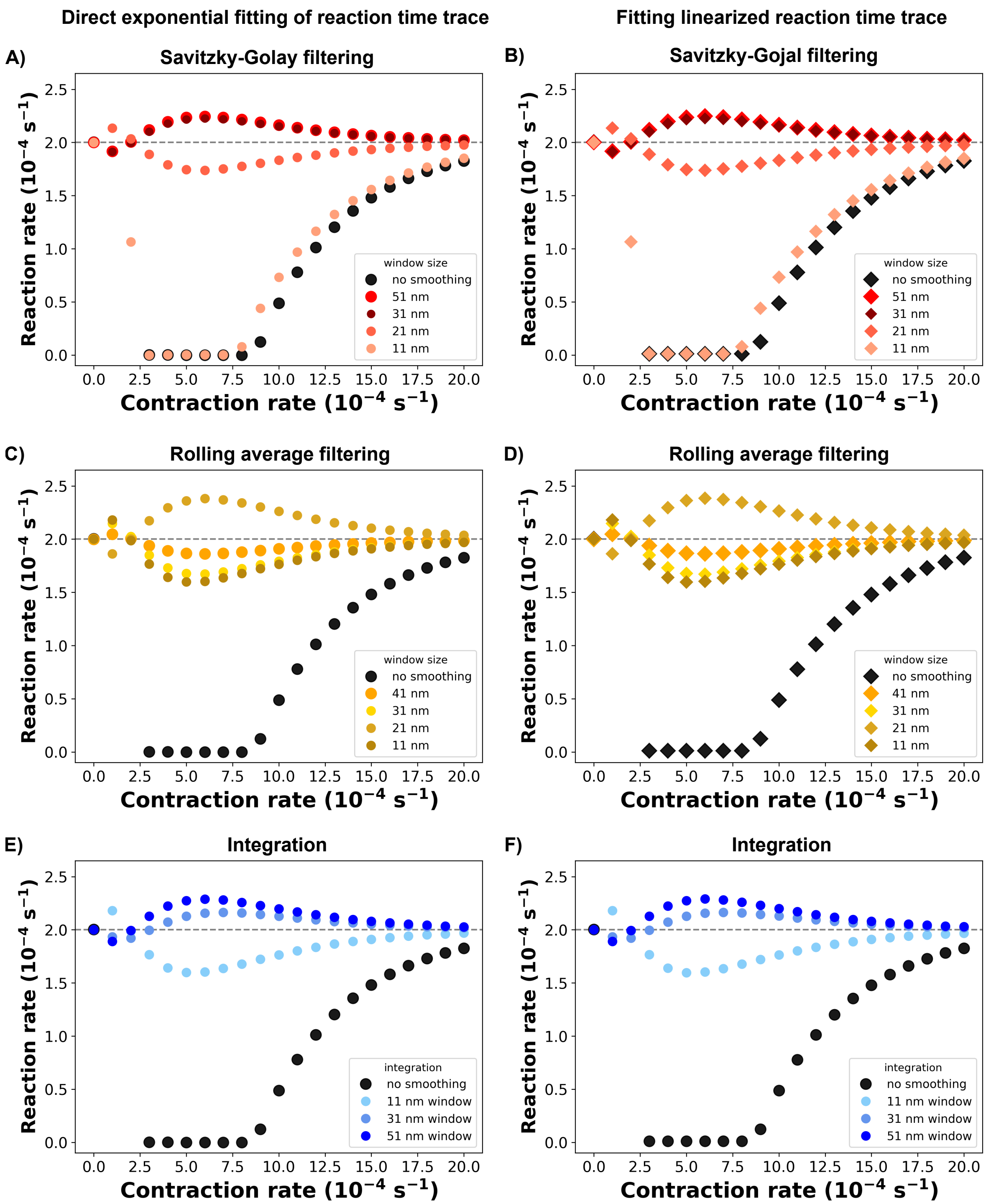


**Figure S7. Data smoothing and integration techniques applied to homogeneous cavities contracting at varying rates.** Comparison of reaction rates obtained by direct exponential fitting of the reaction time trace and by fitting of the linearized reaction trace. Results of the analysis of the unsmoothed data are plotted in black. **A)** and **B)** Comparison for Savitzky-Golay filtering with different filter window sizes. **C)** and **D)** Comparison for rolling average filtering with different filter window sizes. **E)** and **F)** Comparison for spectrum integration on different spectrum windows. Data of simulations with a constant cavity relative contraction = 0.5 % and $k_{reaction} = 2 \times 10^{-4}$ $s^{-1}$. The fit window for both types of fitting was from 2000 to 8000 s.

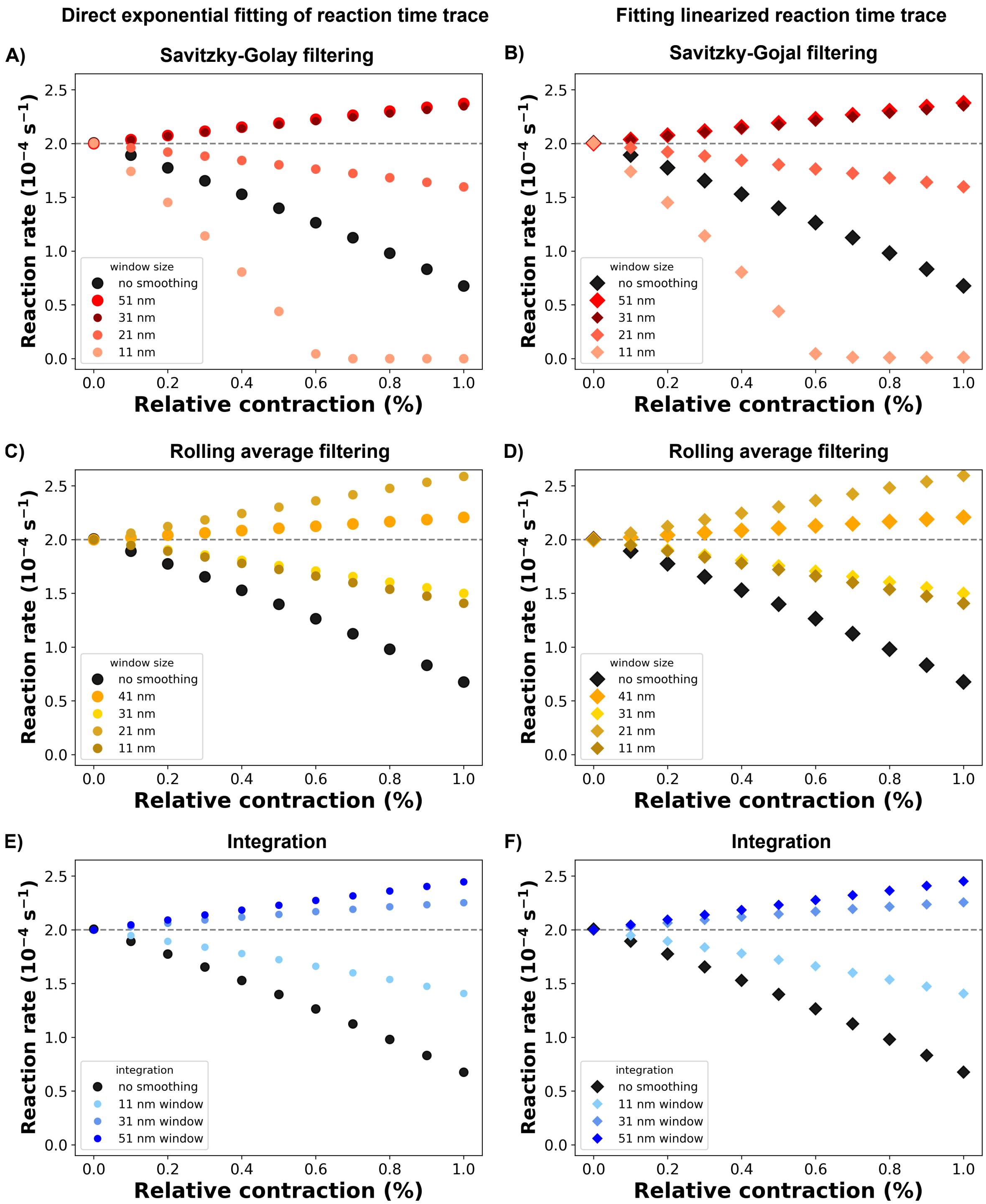


**Figure S8. Data smoothing and integration techniques applied to homogeneous cavities contracting to varying extents.** Comparison of reaction rates obtained by direct exponential fitting of the reaction time trace and by fitting of the linearized reaction trace. Results of the analysis of the unsmoothed data are plotted in black. **A)** and **B)** Comparison for Savitzky-Golay filtering with different filtering window sizes. **C)** and **D)** Comparison for rolling average filtering on different filtering window sizes. **E)** and **F)** Comparison for spectrum integration on different spectral windows. Data of simulations with a constant $k^{cavity}_{contraction} = 9 \times 10^{-4}$ $s^{-1}$ and $k_{reaction} = 2 \times 10^{-4}$ $s^{-1}$. The fit window for both types of fitting was from 2000 to 8000 s.

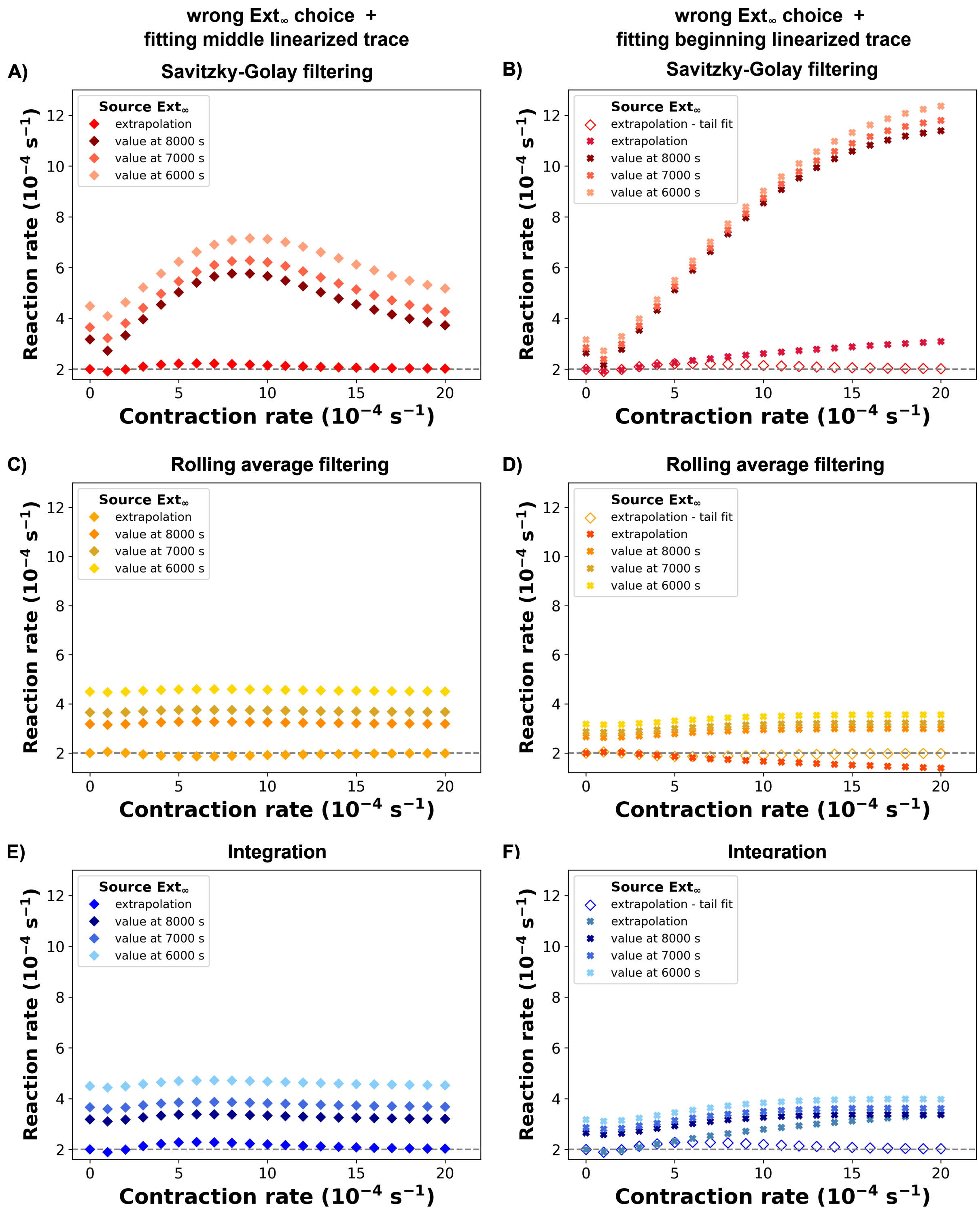


**Figure S9. Combination of data smoothing, $Ext_\infty$ and fit window choice on linearized trace analysis of contracting Fabry-Perot cavities: effect of contraction rate.** After smoothing/integration of the simulated spectra, the linearized time traces were computed using the differential extinction values at 6000, 7000 or 8000 s, instead of using an extrapolated value (see Fig. S7). Fitting was then performed on either the beginning (0-2000 s) or the middle part (2000-4000 s) of the linearized trace. **A)** and **B)** Analysis after Savitzky-Golay filtering (31 point window). **C)** and **D)** Analysis after rolling average filtering (21 point window). **E)** and **F)** Analysis after spectrum integration (400-450 nm region). Data of simulations with a constant cavity relative contraction = 0.5 % and $k_{reaction} = 2 \times 10^{-4}$ $s^{-1}$.

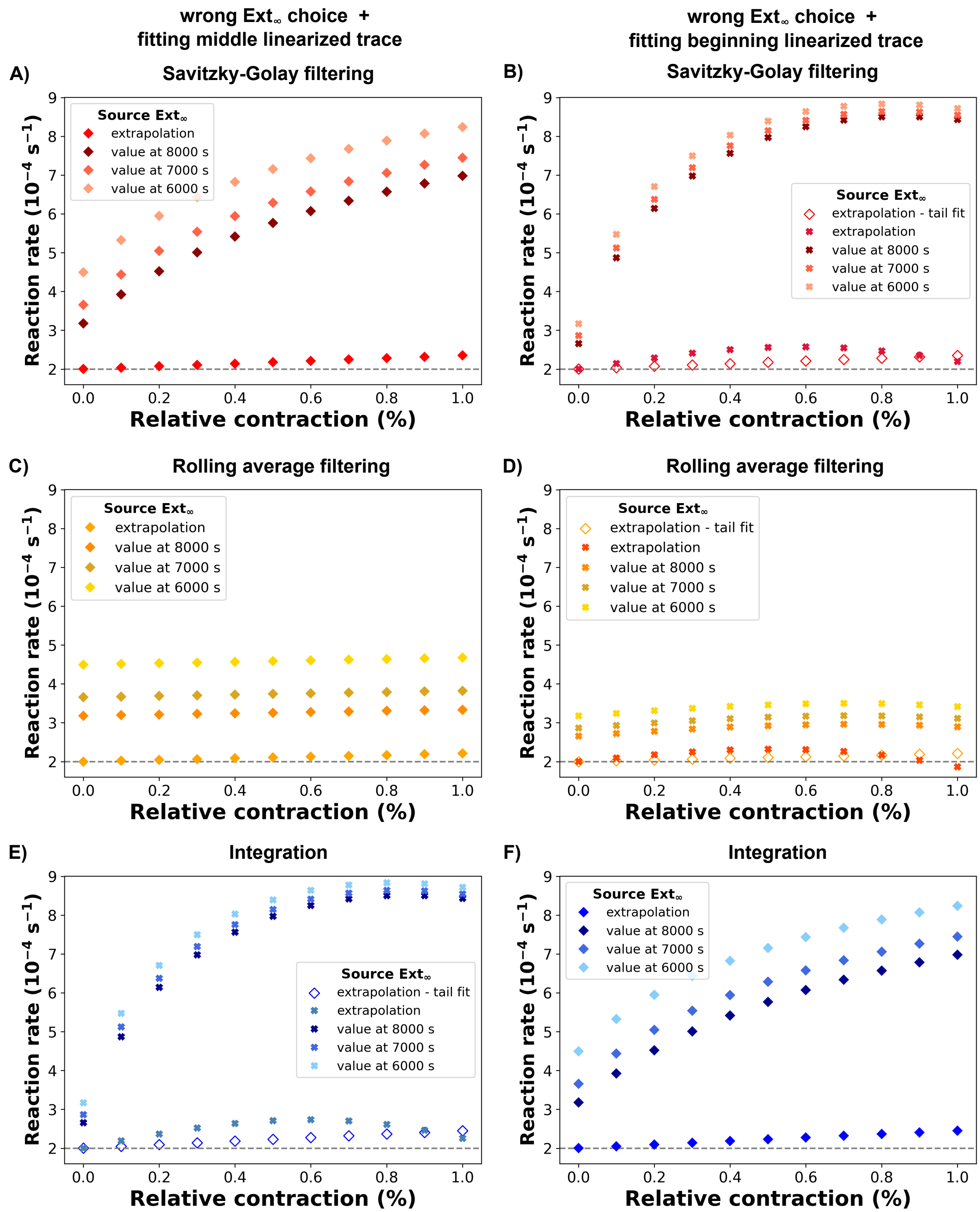


**Figure S10. Combination of data smoothing, $\mathrm{Ext}_\infty$ and fit window choice on linearized trace analysis of contracting Fabry-Perot cavities: effect of relative contraction.** After smoothing/integration of the simulated spectra, the linearized time traces were computed using the differential extinction values at 6000 s, 7000 s or 8000 s, instead of using an extrapolated value (see Fig. S7). Fitting was then performed on either the beginning (0-2000 s) or the middle part (2000-4000 s) of the linearized trace. **A)** and **B)** Analysis after Savitzky-Golay filtering (31 point window). **C)** and **D)** Analysis after rolling average filtering (21 point window). **E)** and **F)** Analysis after spectrum integration (400-450 nm region). Data of simulations with a constant $\mathrm{k}_{\mathrm{contraction}}^{\mathrm{cavity}} = 9 \times 10^{-4}\ \mathrm{s}^{-1}$ and $\mathrm{k}_{\mathrm{reaction}} = 2 \times 10^{-4}\ \mathrm{s}^{-1}$.

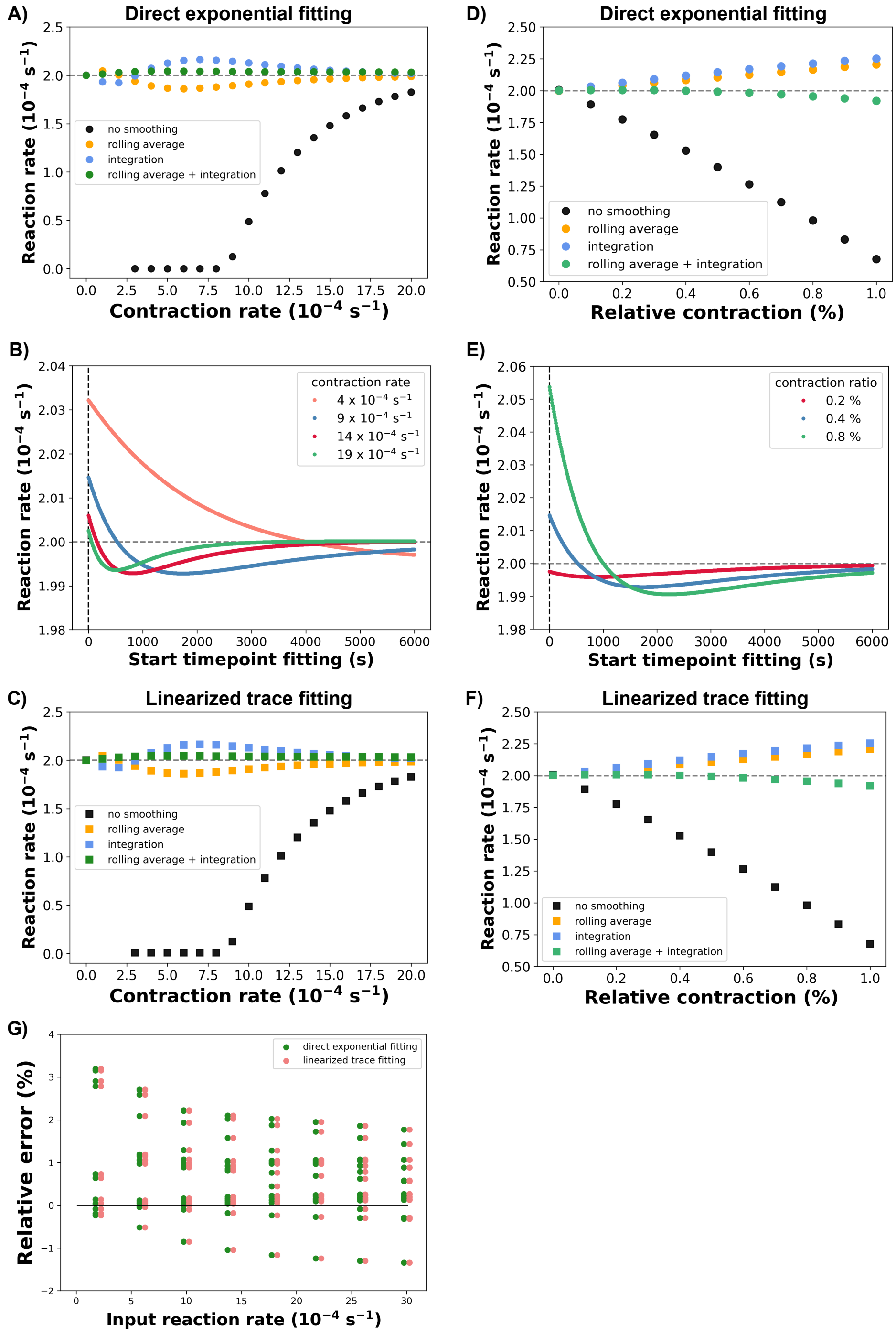


**Figure S11. Performance of integration after rolling average filtering: analysis linearized traces.** **A)** Reaction rates from direct trace fitting vs cavity contraction rate. Cavity relative contraction = 0.5 %. **B)** Influence of starting point fit window on extracted reaction rate for 4 selected cavity contraction rates. **C)** Obtained reaction rates from linearized trace fitting vs cavity contraction rate. Cavity relative contraction = 0.5 %. **D)** Reaction rates from direct trace fitting vs cavity relative contraction. $k^{cavity}_{contraction} = 9 \times 10^{-4}$ s$^{-1}$ **E)** Influence of starting point fit window on extracted reaction rate for 3 selected cavity relative contractions. **F)** Reaction rates from linearized trace fitting vs cavity relative contraction. $k^{cavity}_{contraction} = 9 \times 10^{-4}$ s$^{-1}$ Analysis by applying a rolling average filter on an 41 point window, following by integration of the 410 to 430 nm spectral region.

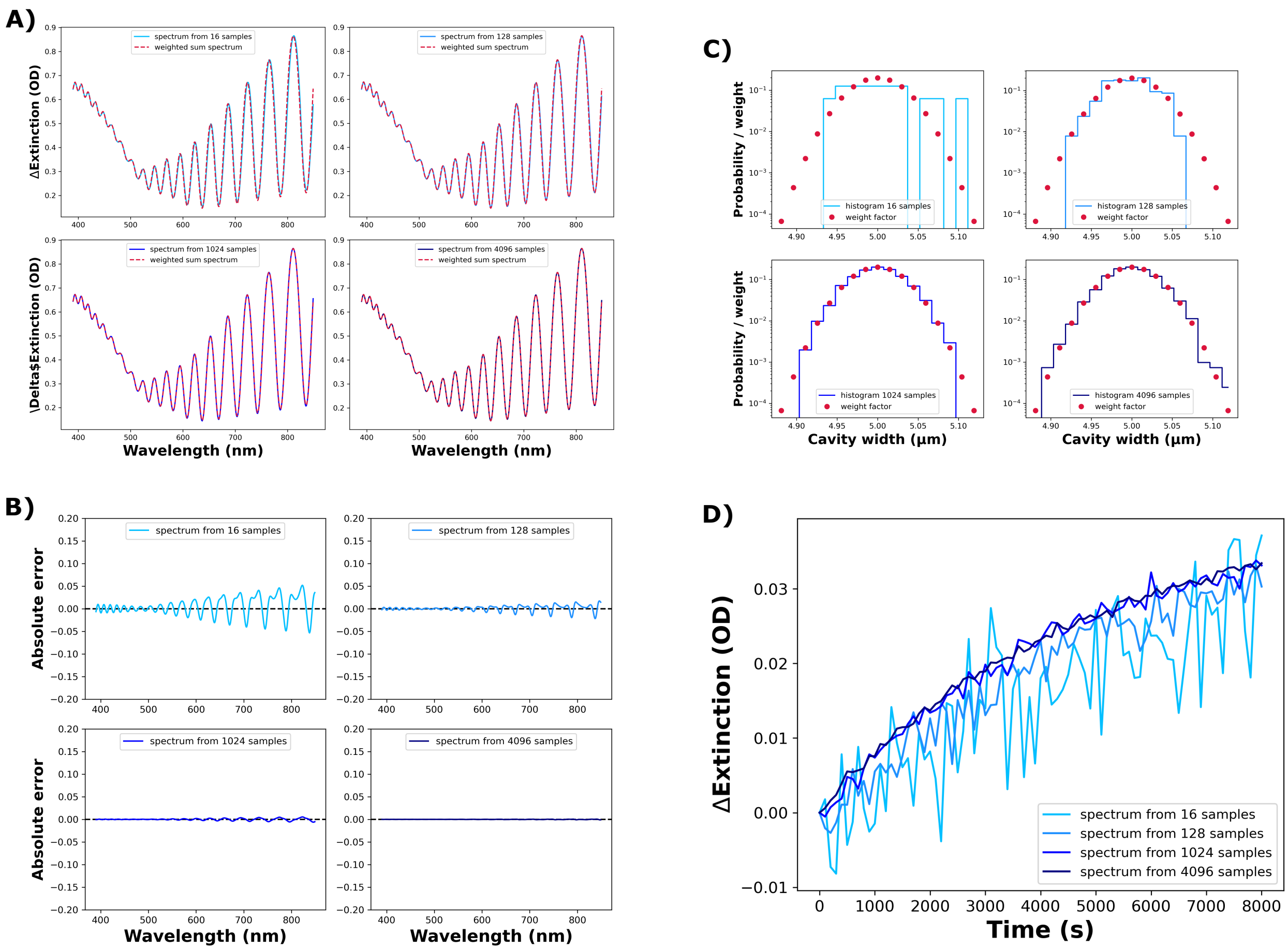


**Figure S12.** Simulating cavity width inhomogeneity by random sampling. **A)** Simulated cavity UV/VIS spectra at t = 0 s. Cyan lines: spectra computed by averaging 16, 128, 1024, 4086 individual spectra. Red lines: reference spectrum computed using the weighted average method.[6] **B)** Absolute differences between the spectra computed by random sampling and a reference spectrum computed by the weighted average method. **C)** Sampled cavity width distributions used for computing the spectra in panel A. **D)** Reaction time traces for an Gaussian inhomogeneous cavity simulated with random cavity width sampling. Parameters: 5 $\mu$m center width and $L_{FWHM}$ = 70 nm.

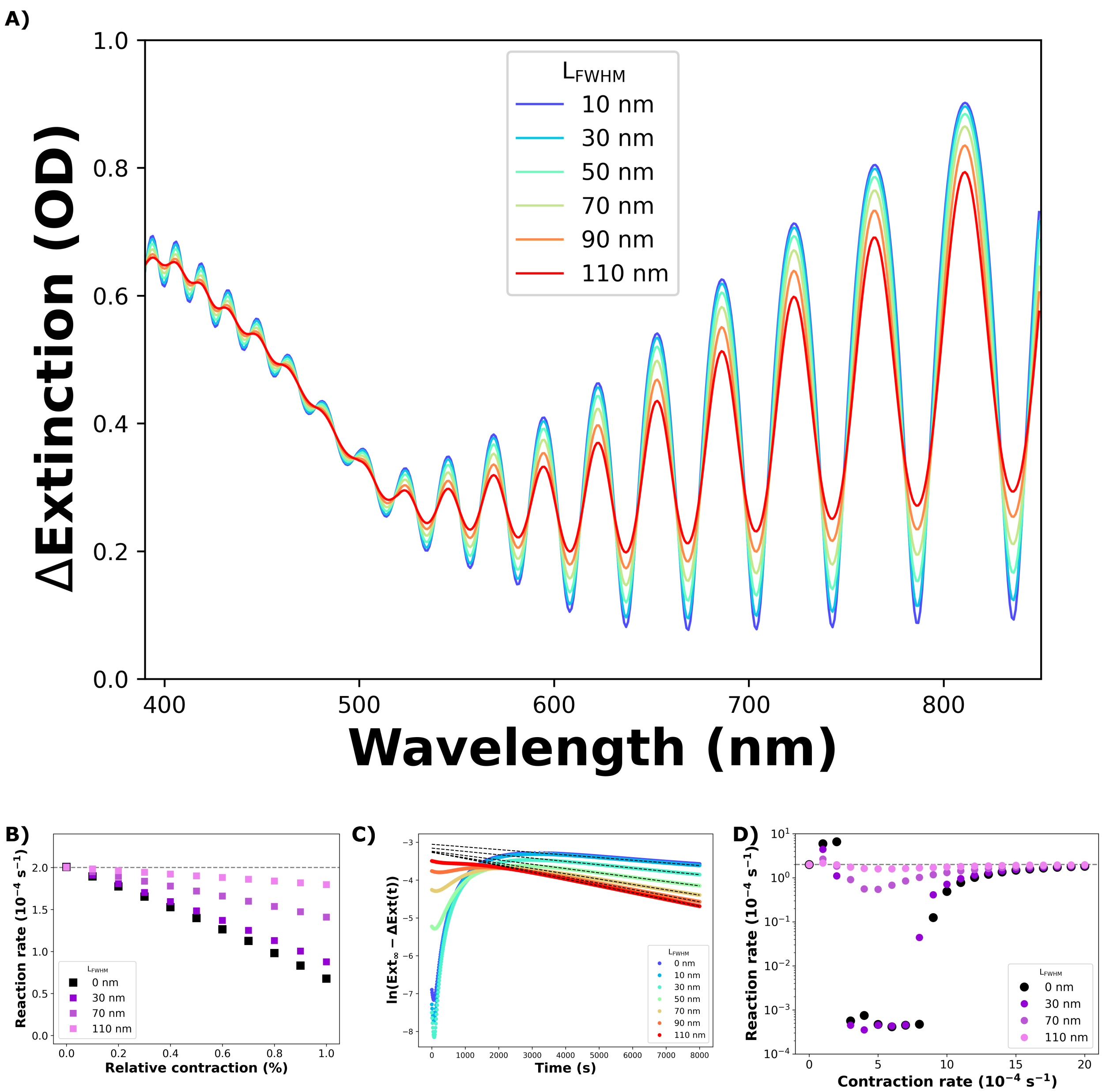


**Figure S13. Inhomogenous cavity with gaussian cavity width broadening: additional data. A)** Full spectra for a Fabry-Perot cavity with varying levels of gaussian cavity width broadening. Medium layer absorption was set to 0 corresponding to $t = 0$ s spectra. **B)** linearized form of the time traces displayed in Figure 5B together with their linear fits (dashed black lines). **C)** and **D)** log plot of obtained reaction rates for cavities contracting with varying contraction rates and exhibiting different levels of gaussian cavity width broadening.

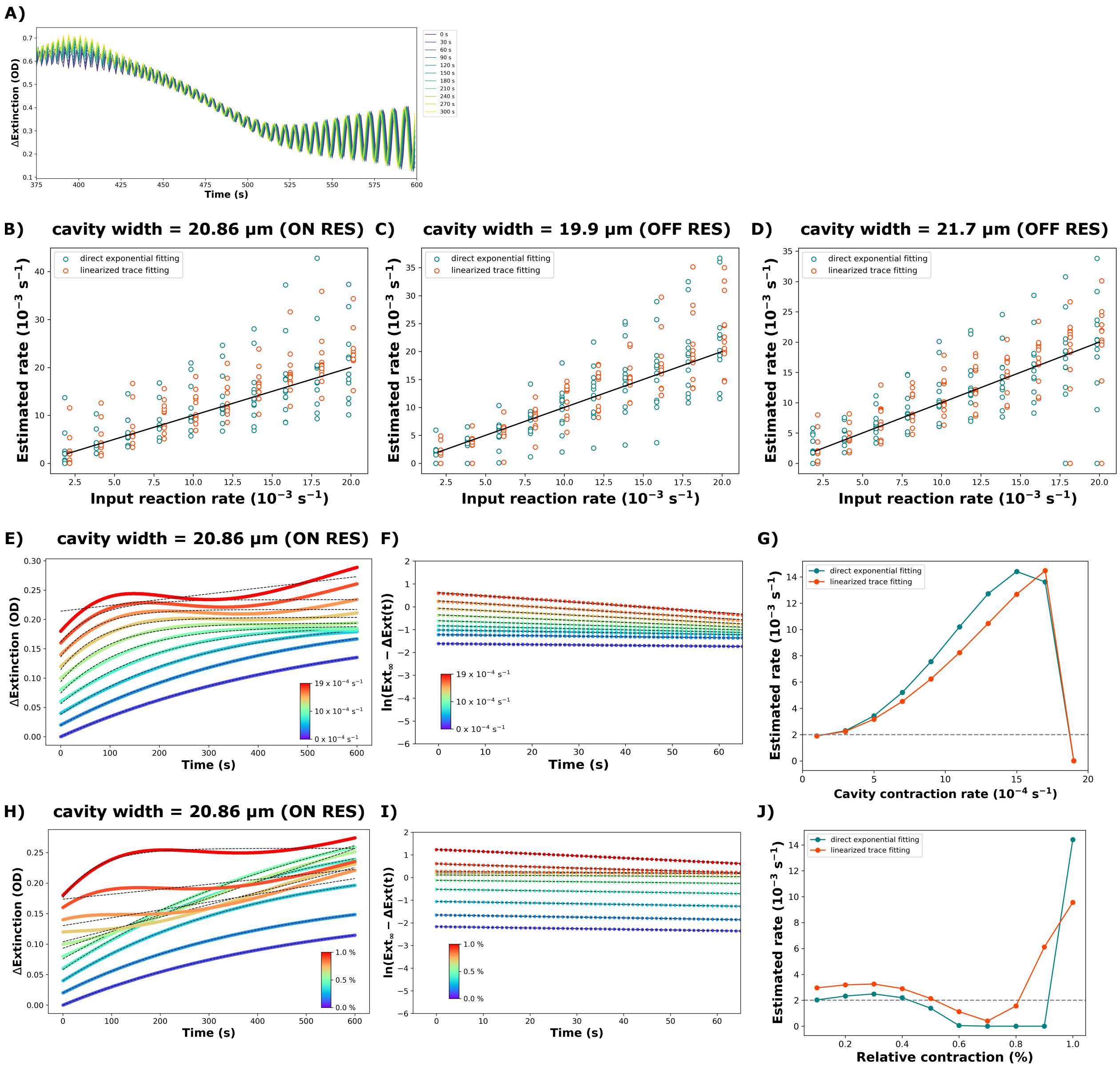


**Figure S14.** Simulating the experimental data of Lather et al. (Angew Chem Intl Ed, 2019).[1] **A)** Example of simulated reaction UV/VIS spectra. **B-D)** Range of possible reaction rates extracted using the analysis method of Lather et al. (Angew Chem Intl Ed, 2019)[1] for cavity widths of B) 20.86 $\mu$m , corresponding to an ON resonance cavity, C) 19.9 $\mu$m and D) 21.7 $\mu$m , both corresponding to OFF resonance cavities. For a given reaction rate, reaction time traces for all combinations of a set of relative contractions (0.2, 0.6 and 1.0%) and contraction rates (2, 8, 14 and $20 \times 10^{-4}$ s−1) were simulated and analyzed by fitting both the raw and linearized traces (teal and orangered curves resp.). Each dot thus represents a simulation of a cavity with one set of contraction parameters (*i.e.* rel. ratio and rate). **E)** and **F)** Raw and linearized reaction time traces for cavities contracting 1 % at varying rates. **G)** Plot of the reaction rates obtained from analysis of the time traces in E) (teal) and F) (orangered). **H)** and **I)** Raw and linearized reaction time traces for cavities contracting to varying degrees at a rate of $15 \times 10^{-4}$ s$^{-1}$. **J)** Plot of the reaction rates obtained from analysis of the time traces in H) (teal) and I) (orangered). Simulations in E-J) with a input reaction rate $k_{reaction} = 2 \times 10^{-3}$ s$^{-1}$.

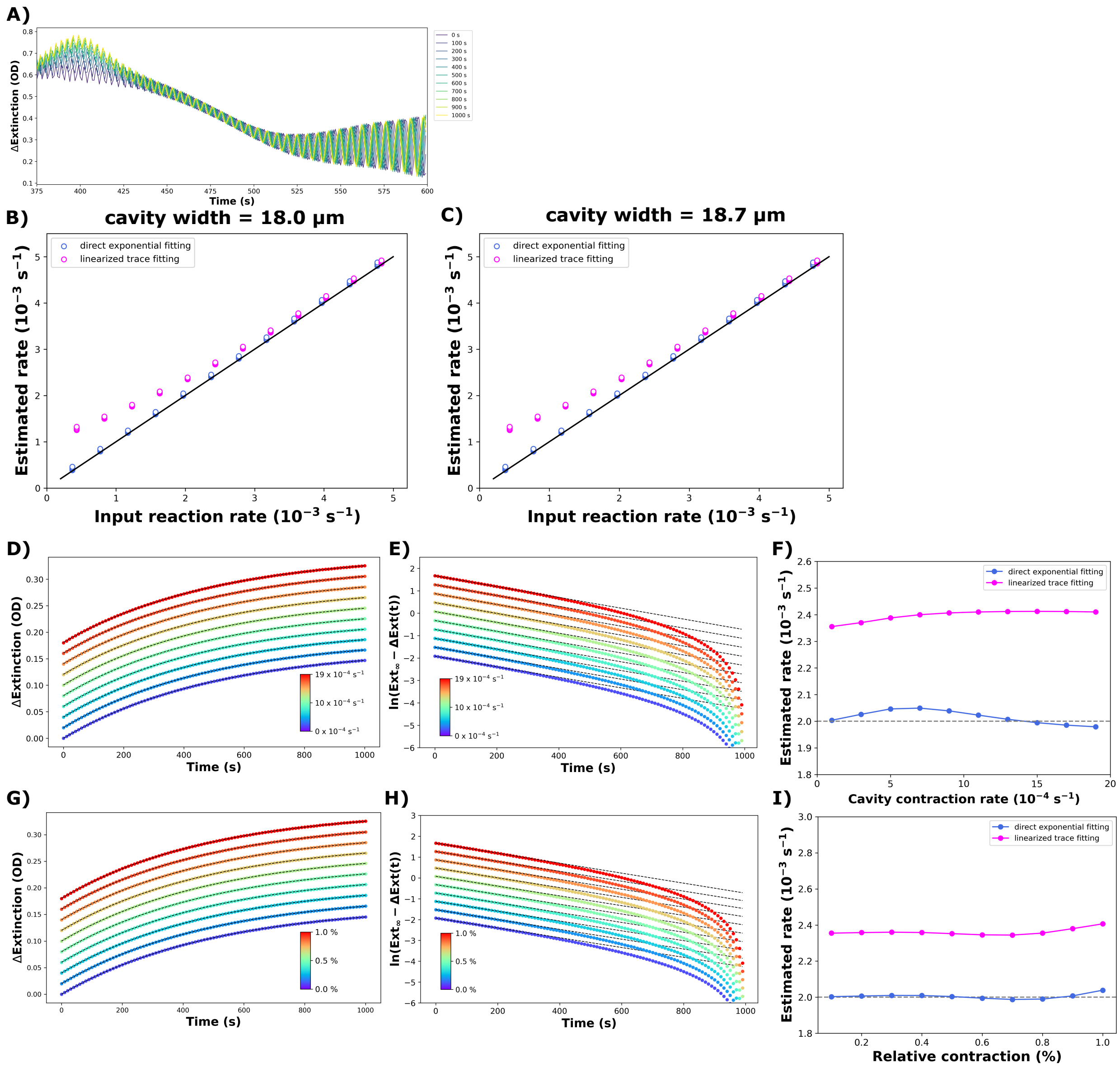


**Figure S15.** Simulating the experimental data of Singh et al. (ChemPhysChem, 2023).[2] **A)** Example of simulated reaction UV/VIS spectra. **B)** & **C)** Range of possible reaction rates extracted using the analysis method of Singh et al. (ChemPhysChem, 2023)[2] for cavity widths of B) 18.0 $\mu$m and C) 18.7 $\mu$m . For each reaction rate, reaction time traces for all combinations of a set of relative contractions (0.2, 0.6 and 1.0%) and contraction rates ($2, 8, 14$ and $20 \times 10^{-4}$ s$-1$) were simulated and analyzed by fitting both the raw and linearized traces (blue and pink curves resp.). Each dot thus represents a simulation of a cavity with one set of contraction parameters (*i.e.* rel. ratio and rate). **D)** and **E)** Raw and linearized reaction time traces for cavities contracting 1 % at varying rates. **F)** Plot of the reaction rates obtained from analysis of the time traces in C) (blue) and D) (pink). **G)** and **H)** Raw and linearized reaction time traces for cavities contracting to varying degrees at a rate of $15 \times 10^{-4}$ s$^{-1}$. **I)** Plot of the reaction rates obtained from analysis of the time traces in F) (blue) and G) (pink). Simulations in D-I) with a input reaction rate $\mathrm{k_{reaction}} = 2 \times 10^{-3}$ s$^{-1}$.

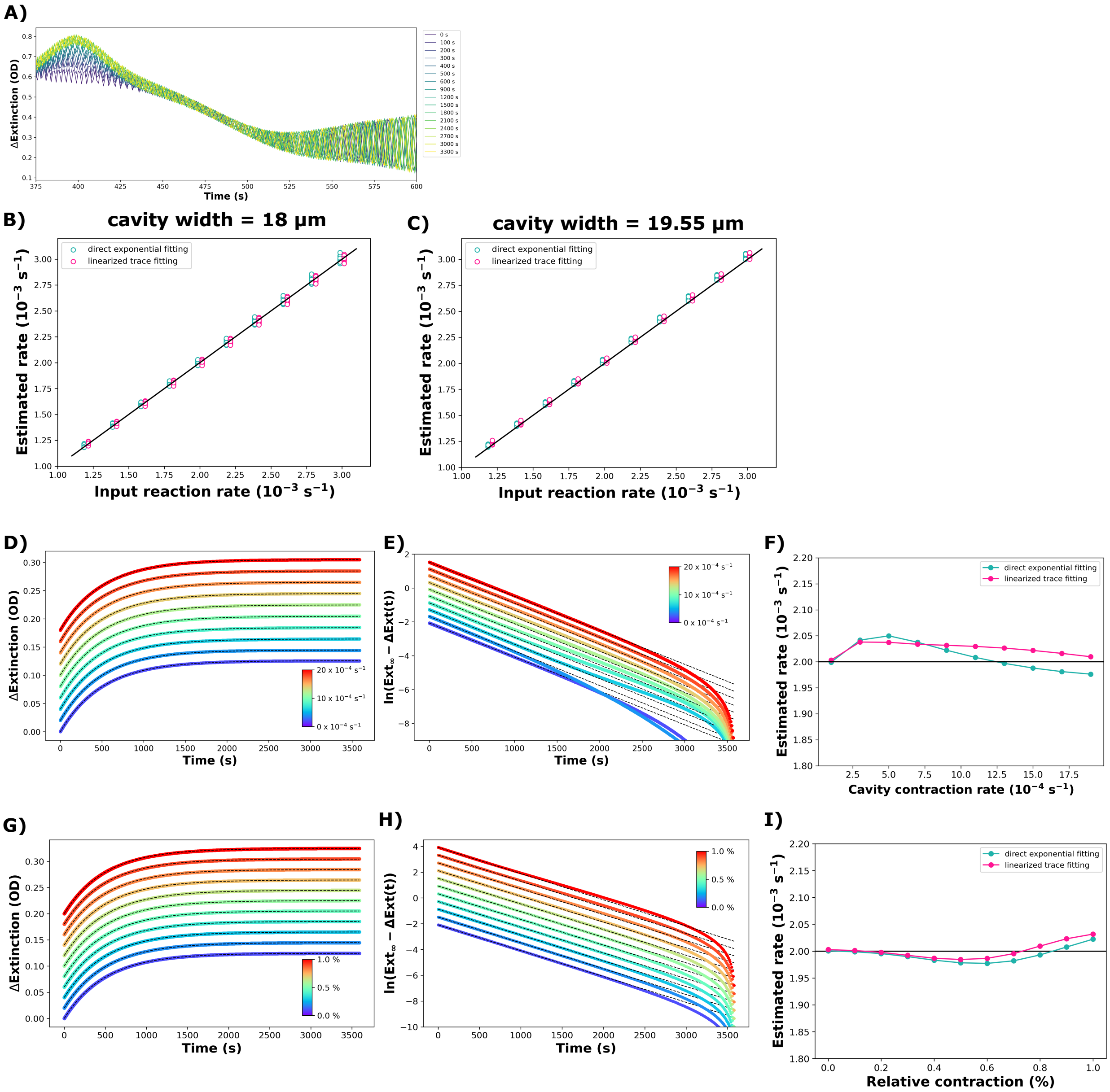


**Figure S16.** Simulating the experimental data of Weisehan and Xiong (J Chem Phys, 2021).[3] **A)** Example of simulated reaction UV/VIS spectra. **B)** & **C)** Range of possible reaction rates extracted using the analysis method of Weisehan and Xiong (J Chem Phys, 2021)[3] for cavity widths of B) 18.0 $\mu$m and C) 19.55 $\mu$m . For each reaction rate, reaction time traces for all combinations of a set of relative contractions (0.2, 0.6 and 1.0%) and contraction rates (2, 8, 14 and $20 \times 10^{-4}$ s−1) were simulated and analyzed by fitting both the raw and linearized traces (green and pink curves resp.). Each dot thus represents a simulation of a cavity with one set of contraction parameters (*i.e.* rel. ratio and rate). **D)** and **E)** Raw and linearized reaction time traces for cavities contracting 1 % at varying rates. **F)** Plot of the reaction rates obtained from analysis of the time traces in C) (green) and D) (pink). **G)** and **H)** Raw and linearized reaction time traces for cavities contracting to varying degrees at a rate of $15 \times 10^{-4}$ s$^{-1}$. **I)** Plot of the reaction rates obtained from analysis of the time traces in F) (green) and G) (pink). Simulations in D-I) with a input reaction rate $k_{reaction} = 2 \times 10^{-3}$ s$^{-1}$.

# References


(1) Lather, J.; Bhatt, P.; Thomas, A.; Ebbesen, T. W.; George, J. Cavity Catalysis by Cooperative Vibrational Strong Coupling of Reactant and Solvent Molecules. *Angewandte Chemie International Edition* **2019**, *58*, 10635–10638.

(2) Singh, J.; Lather, J.; George, J. Solvent Dependence on Cooperative Vibrational Strong Coupling and Cavity Catalysis. *ChemPhysChem* **2023**, *24*, e202300016.

(3) Wiesehan, G. D.; Xiong, W. Negligible rate enhancement from reported cooperative vibrational strong coupling catalysis. *The Journal of Chemical Physics* **2021**, *155*, 241103.

(4) Saleh, B. E. A.; Teich, M. C. *Fundamentals of Photonics*, 3rd ed.; Wiley-Interscience, Inc., 2019.

(5) Rosenblatt, G.; Simkhovich, B.; Bartal, G.; Orenstein, M. Nonmodal Plasmonics: Controlling the Forced Optical Response of Nanostructures. *Phys. Rev. X* **2020**, *10*, 011071.

(6) Michon, M. A.; Simpkins, B. S. Impact of Cavity Length Non-uniformity on Reaction Rate Extraction in Strong Coupling Experiments. *Journal of the American Chemical Society* **2024**, *146*, 30596–30606, PMID: 39466594.

(7) Orosco, J.; Coimbra, C. F. M. On a causal dispersion model for the optical properties of metals. *Applied Optics* **2018**, *57*, 5333–5347.

(8) Virtanen, P. et al. SciPy 1.0: Fundamental Algorithms for Scientific Computing in Python. *Nature Methods* **2020**, *17*, 261–272.
